# A Dual-Ligand PDC Co-Targeting FOLR1 and c-Met Enhances Tumor Accumulation and Antitumor Efficacy in Ovarian Cancer

**DOI:** 10.3390/cancers18183013

**Published:** 2026-09-17

**Authors:** Honghong Cai, Tian Tao, Yuan Gao, Xiuwu Tang, Youguo Chen, He Zhang

**Affiliations:** 1 Department of Obstetrics and Gynecology, The First Affiliated Hospital of Soochow University, Suzhou 215005, China; hhcai824@alu.suda.edu.cn (H.C.); taotiantt@suda.edu.cn (T.T.); tangxiuwu@suda.edu.cn (X.T.); 2 Department of Pharmacy, The First Affiliated Hospital of Soochow University, Suzhou 215005, China; gaoyuan29@suda.edu.cn

**Keywords:** epithelial ovarian cancer (EOC), peptide-drug conjugate (PDC), dual-targeting therapy, FOLR1/c-Met

## Abstract

**Background**: Epithelial ovarian cancer (EOC) remains the most lethal gynecological malignancy. Single-targeted ADCs/PDCs are limited by “on-target, off-tumor” toxicity. We developed FMM-66, a dual-ligand peptide-drug conjugate targeting FOLR1 and c-Met, to improve tumor-selective drug delivery. **Methods**: FMM-66 was rationally designed by integrating FOLR1- and c-Met-targeting peptide ligands into a single PDC architecture. Binding and cellular internalization assays were performed in ovarian cancer cell lines with different FOLR1/c-Met expression profiles, as well as in engineered overexpression cell models, to comprehensively evaluate the targeting properties of FMM-66. Antitumor efficacy, intratumoral pharmacokinetics, and biodistribution were evaluated in female BALB/c nude mice bearing Caov3 xenografts, with the single-ligand PDCs FA-MMAE and GE137-MMAE as comparators. Normal-tissue biodistribution and systemic safety were further assessed in female Sprague–Dawley rats. **Results**: FMM-66 demonstrated enhanced binding affinity and significantly increased cellular internalization compared with single-ligand PDCs in vitro. Receptor-blocking experiments confirmed contributions from both FOLR1 and c-Met, whereas internalization was driven predominantly by FOLR1. In vivo studies revealed potent tumor growth inhibition and superior tumor accumulation in FOLR1/c-Met double-positive ovarian tumors than the single-ligand comparators. Furthermore, FMM-66 exhibited rapid systemic clearance while released MMAE showed prolonged intratumoral exposure. At the therapeutic-equivalent dose in rats, no significant biochemical abnormalities or body-weight loss were observed; transient ALT, AST, creatinine, and urea elevations at the higher dose largely resolved by Day 14. **Conclusions**: FMM-66 demonstrated enhanced tumor accumulation and potent antitumor efficacy, with preliminary tolerability at the tested dose levels. These findings support dual-ligand targeting as a promising strategy for improving tumor-selective payload delivery and warrant further preclinical evaluation of FMM-66.

## Simple Summary

Ovarian cancer is one of the deadliest cancers in women. Newer targeted treatments can deliver toxic drugs directly to tumors, but they often damage healthy organs because the markers used to identify cancer cells also exist in normal tissues. This safety issue causes severe side effects and limits treatment effectiveness. In this study, researchers developed a molecule designed to simultaneously target FOLR1 and c-Met—two proteins highly expressed on ovarian cancer cells—ensuring efficient delivery of the cell-killing payload. FMM-66 showed strong tumor accumulation and antitumor activity while being rapidly cleared from the bloodstream. Single-dose studies also showed good tolerability at the therapeutic-equivalent dose, although transient liver and kidney biomarker elevations occurred at a higher dose. These findings support FMM-66 as a promising preclinical dual-targeting strategy for ovarian cancer.

## 1. Introduction

Epithelial ovarian cancer (EOC) remains the most lethal gynecological malignancy worldwide. Despite advances in cytoreductive surgery and platinum-based chemotherapy, approximately 70% of patients experience disease recurrence and ultimately develop platinum-resistant disease, for which therapeutic options remain extremely limited. Consequently, the 5-year overall survival rate has remained at approximately 47% over the past decade [1,2,3]. These clinical challenges underscore the urgent need for precision therapeutic strategies that selectively target molecular vulnerabilities in ovarian cancer.

Folate receptor alpha (FOLR1) has emerged as one of the most attractive therapeutic targets for EOC [4,5,6]. FOLR1 is a glycosylphosphatidylinositol (GPI)-anchored membrane protein responsible for folate uptake, thereby supporting nucleotide biosynthesis and cellular proliferation [7,8]. In normal tissues, FOLR1 expression is largely restricted to the apical surface of polarized epithelial cells, whereas approximately 80% of high-grade serous ovarian carcinomas exhibit high FOLR1 expression [6,7,9,10]. This tumor-selective expression profile has facilitated the development of FOLR1-targeted antibody–drug conjugates (ADCs). Mirvetuximab soravtansine (Elahere), consisting of a humanized anti-FOLR1 monoclonal antibody conjugated to the microtubule inhibitor DM4 through a cleavable linker, received accelerated FDA approval in November 2022 and was converted to full approval in March 2024 as the first ADC for platinum-resistant ovarian cancer with high FOLR1 expression [11,12]. In addition, several next-generation FOLR1-targeted ADCs, including ZW191, BAT8006, IMGN151, and LY4170156, are currently undergoing clinical evaluation [13,14,15,16].

The mesenchymal–epithelial transition factor (c-Met), encoded by the MET proto-oncogene, is another clinically relevant target that contributes to tumor proliferation, invasion, metastasis, and therapeutic resistance in multiple solid tumors, including ovarian cancer [17,18]. GE137, a 26-amino acid bicyclic peptide, binds the extracellular domain of c-Met with high affinity without competing with its endogenous ligand, hepatocyte growth factor (HGF) [19]. Owing to its small molecular size, GE137 exhibits favorable pharmacokinetic properties, including rapid renal clearance, low background tissue uptake, and minimal immunogenicity, representing potential advantages over conventional antibody-based targeting agents [20]. Phase I clinical studies involving healthy volunteers and cancer patients further demonstrated that GE137 was well tolerated at doses up to 0.36 mg/kg without clinically significant adverse events [21]. However, GE137 alone does not induce c-Met activation or receptor internalization, presenting a challenge for the development of c-Met-targeted drug conjugates that rely on efficient intracellular payload delivery [22].

Although ADCs and peptide-drug conjugates (PDCs) have emerged as promising precision therapeutics, their clinical efficacy remains constrained by a fundamental limitation known as “on-target, off-tumor” toxicity. This limitation arises because many tumor-associated antigens are overexpressed rather than exclusively expressed in tumor tissues. Consequently, target engagement in normal tissues leads to unintended payload internalization and cytotoxicity, thereby narrowing the therapeutic window and limiting the maximum tolerated dose [23,24]. While peptide-based conjugates generally exhibit faster systemic clearance than ADCs, single-targeted PDCs remain susceptible to this intrinsic pharmacological challenge.

Dual-targeting strategies have recently attracted increasing attention as a potential solution to improve tumor selectivity by simultaneously recognizing two independently expressed tumor-associated receptors [25,26,27]. Such an approach is expected to enhance tumor accumulation through cooperative binding while reducing off-tumor exposure in normal tissues expressing only one target receptor. Nevertheless, dual-targeted PDCs for ovarian cancer remain largely unexplored.

In the present study, we developed FMM-66, a novel dual-ligand peptide-drug conjugate simultaneously targeting FOLR1 and c-Met. We systematically evaluated its binding affinity, receptor-mediated internalization, pharmacokinetic behavior, antitumor efficacy, biodistribution, and safety in vitro and in vivo. Compared with single-ligand PDCs, FMM-66 demonstrated enhanced tumor accumulation and superior antitumor activity in FOLR1/c-Met co-expressing ovarian cancer models while reducing systemic exposure to normal tissues through rapid clearance. These findings provide preclinical proof of concept for a dual-targeting PDC strategy designed to improve tumor-selective drug delivery.

## 2. Materials and Methods

### 2.1. Cell Lines and Cell Culture

Human ovarian cancer cell lines Caov-3 (ATCC, HTB-75), COV434 (ECACC, 07071909), SKOV3 (ATCC, HTB-77), and A2780 (ECACC, 93112519) were obtained from Sunncell (Wuhan, China). JHOS-4 (Cellosaurus, CVCL_4649), DOV13 (Cellosaurus, CVCL_6774) and HEK293T (ATCC, CRL-3216) were purchased from Procell (Wuhan, China). Cells were maintained in a humidified incubator at 37 °C with 5% CO_2_. Caov-3, A2780, COV434, and HEK293T were cultured in DMEM supplemented with 10% fetal bovine serum (FBS) and 1% penicillin/streptomycin (P/S). SKOV3 was maintained in McCoy’s 5A medium with 10% FBS and 1% P/S. JHOS-4 was cultured in DMEM/F-12 containing 10% FBS and 1% P/S, while DOV13 was maintained in RPMI-1640 supplemented with 10% FBS and 1% P/S. All cell lines were authenticated by short tandem repeat (STR) profiling and confirmed to be mycoplasma-free prior to experimentation.

### 2.2. Compounds

FMM-66, GE137-MMAE, FA-MMAE, FMM-66-Cy5, GE137-Cy5, and FA-Cy5 were custom synthesized by Kameiluo Co., Ltd. (Shanghai, China). The identity of each compound was confirmed by LC–MS, and chromatographic purity was determined by analytical HPLC. All compounds exhibited a purity of >95%. Representative HPLC chromatograms and LC–MS spectra are provided in Figures S1–S6. The chemical structures of these compounds are shown in Figure S7.

### 2.3. Surface Plasmon Resonance (SPR) Analysis

SPR assays were performed at 25 °C using a Biacore S200 instrument (Cytiva, Marlborough, MA, USA). HBS-EP+ buffer (10 mM HEPES, 150 mM NaCl, 3 mM EDTA, and 0.05% *v*/*v* Surfactant P20, pH 7.4) was used as the running buffer throughout the experiments. Recombinant target proteins carrying a C-terminal Avi-tag were biotinylated in vitro using BirA biotin ligase according to the manufacturer’s instructions and immobilized onto a Series S Sensor Chip SA (Cytiva) via streptavidin–biotin capture. Test compounds were serially diluted in running buffer to generate a seven-point concentration series (5–320 nM) and injected in randomized order at a flow rate of 30 μL/min with an association time of 120 s followed by a dissociation phase of 180 s. Binding kinetics were analyzed using Biacore S200 Evaluation Software v1.0 with a 1:1 kinetic binding model.

### 2.4. Cell Binding, Competition, and Internalization Assays

Cell binding affinity (EC_50_), receptor specificity, and internalization of Cy5-labeled compounds were evaluated by flow cytometry. FOLR1- and/or c-Met-positive ovarian cancer cells, together with receptor-negative control cells, were harvested using enzyme-free cell dissociation buffer and washed twice with ice-cold Hanks’ balanced salt solution (HBSS) supplemented with 1% bovine serum albumin (BSA). Cells were incubated with serial dilutions of Cy5-labeled compounds for 30 min at 4 °C to prevent receptor-mediated endocytosis. Following two washes with binding buffer, mean fluorescence intensity (MFI) was determined by flow cytometry, and EC_50_ values were calculated by nonlinear regression analysis.

For competitive binding assays, Caov3 cells were pre-incubated with 10 nM FA-Cy5 or 50 nM GE137-Cy5 on ice, followed by incubation with increasing concentrations of FMM-66 or the corresponding single-ligand conjugate (FA-MMAE or GE137-MMAE) for 30 min at 4 °C. After washing, residual Cy5 fluorescence was quantified by flow cytometry to evaluate competitive receptor binding.

Receptor-mediated internalization was assessed using an acid-stripping assay. Cells were first incubated with 200 nM Cy5-labeled compounds for 30 min at 4 °C to achieve surface binding, and then transferred to 37 °C for 0.5, 1, 2, 4, 8, 16, or 24 h to permit internalization. Surface-bound ligands were removed by treatment with 0.2 M glycine-HCl (pH 2.5) for 5 min on ice and immediately neutralized with 0.5 M phosphate buffer (pH 7.4). The remaining fluorescence, representing internalized compounds, was quantified by flow cytometry. Internalization efficiency was calculated as the percentage of acid-resistant fluorescence relative to total cell-associated fluorescence: Internalization (%) = (MFI (acid-treated)/MFI (total)) × 100.

Flow cytometric analysis was performed using a BD FACSCanto II flow cytometer (BD Biosciences, San Jose, CA, USA). At least 10,000 events were acquired for each sample, and data were analyzed using FlowJo software (v10.0.7, Tree Star Inc., Ashland, OR, USA).

Surface expression of FOLR1 and c-Met was additionally quantified by flow cytometry in Caov3, SKOV3, JHOS4, DOV13, A2780, and COV434 cells, and mean fluorescence intensity (MFI) was used to compare relative receptor expression among cell lines. For receptor-blocking experiments, Caov3 cells were incubated with vehicle, 10 μM folate to block FOLR1, 1 mg/mL telisotuzumab to block c-Met, or the combination of folate and telisotuzumab before exposure to increasing concentrations of FMM-66-Cy5. Cell-associated fluorescence was quantified by flow cytometry as described above.

### 2.5. Cell Viability Assay

Cells were seeded into 96-well plates and allowed to attach overnight. The following day, cells were treated with nine-point serial dilutions of the indicated compounds (1.5–10,000 nM) for 72 h. Cell viability was then determined using the CellTiter-Glo Luminescent Cell Viability Assay (Promega, Madison, WI, USA) according to the manufacturer’s instructions. Briefly, culture medium was removed, and 100 μL of CellTiter-Glo reagent was added to each well. Luminescence was measured using a SynergyMx microplate reader (BioTek Instruments, Winooski, VT, USA). Half-maximal inhibitory concentration (IC_50_) values were calculated by nonlinear regression using GraphPad Prism (v7.0, GraphPad Software, San Diego, CA, USA). To examine the contribution of cathepsin B to FMM-66-mediated cytotoxicity, Caov3 cells were treated with FMM-66 in the presence or absence of the cathepsin B inhibitor CA-074 (5 nM). CA-074 alone was also evaluated for effects on cell viability under the same assay conditions.

### 2.6. Linker Cleavage Assay

The enzymatic cleavage of the Val-Cit linker in FMM-66 was evaluated using recombinant human cathepsin B (Sigma-Aldrich, Darmstadt, Germany; catalog no. 219362). FMM-66 was prepared as a 1.5 mg/mL stock solution in 1 mM EDTA and diluted to a final concentration of 50 μg/mL for the assay. The cleavage reaction was initiated by the addition of human cathepsin B (200 mU/mL) and incubated under the specified conditions. Aliquots were collected at 0, 4, 8, 16, 24, 36, and 48 h for quantitative determination of intact FMM-66 and released monomethyl auristatin E (MMAE) by liquid chromatography–tandem mass spectrometry (LC–MS/MS). Quantification was performed using calibration curves, and chromatographic integration and data analysis were carried out with Analyst^®^ software (v1.7.2, SCIEX, Framingham, MA, USA).

### 2.7. In Vivo Antitumor Efficacy Study

All animal experiments were conducted in accordance with the guidelines of the Institutional Animal Care and Use Committee (IACUC) and approved by the Ethics Committee of the Medical College of Soochow University (Approval No. IACUC-202409A0456). Female BALB/c nude mice (6–8 weeks old, 18–22 g) were purchased from MODEL Organisms Co., Ltd. (Shanghai, China). Subcutaneous xenograft tumors were established by injecting 5 × 10^6^ Caov3 cells suspended in 100 μL PBS/Matrigel (1:1) into the right flank of each mouse. When tumor volumes reached approximately 200 mm^3^, 24 mice (*n* = 6 per group) were randomly assigned to treatment groups and received intravenous administration equimolar doses (0.5 μmol/kg) of FMM-66 (2.5 mg/kg), FA-MMAE (1.0 mg/kg), or GE137-MMAE (2.25 mg/kg) via the lateral tail vein once weekly for three consecutive weeks (QW × 3). Tumor dimensions and body weights were recorded twice weekly throughout the treatment period. Tumor volume (V) was calculated using the modified ellipsoid formula: V = 0.5 × L × W^2^, where L represents the longest tumor diameter and W represents the perpendicular tumor diameter. Tumor growth inhibition (TGI) was calculated at the study endpoint.

### 2.8. Pharmacokinetic and Biodistribution Analysis

The pharmacokinetic and tissue distribution profiles of FMM-66, FA-MMAE, and GE137-MMAE were evaluated in Caov3 tumor-bearing BALB/c nude mice. To compare the intratumoral pharmacokinetics of the three PDCs, 45 tumor-bearing mice were randomly assigned to three treatment groups (n = 15 per group) and received a single intravenous administration of FMM-66 (2.5 mg/kg), FA-MMAE (1.0 mg/kg), or GE137-MMAE (2.25 mg/kg). Tumor tissues were collected at 1, 2, 6, 24, 96, and 168 h after administration (n = 3 per time point). At 24 h, major normal tissues were additionally collected for comparative tissue distribution analysis.

To further characterize the pharmacokinetic profiles of intact FMM-66 and its released payload MMAE in the circulation and tumor tissue, Caov3 tumor-bearing mice received a single intravenous dose of FMM-66 (2.5 mg/kg). Blood and tumor samples were collected at 0.5, 1, 2, 6, 24, 96, and 168 h after administration (n = 3 per time point). Plasma was obtained by centrifugation of blood samples at 5000× *g* for 10 min at 4 °C and stored until analysis.

Tumor and normal tissue samples were weighed and homogenized in saline at a tissue weight-to-homogenization volume ratio of 1:4 (*w*/*v*). Tissue homogenates were centrifuged at 4700 rpm for 15 min at 4 °C, and the resulting supernatants were collected for quantitative analysis. FMM-66, FA-MMAE, GE137-MMAE, and released MMAE were quantified using validated liquid chromatography–tandem mass spectrometry (LC–MS/MS) methods. A 5 μL aliquot of each prepared sample was injected into a triple-quadrupole tandem mass spectrometer (5500 QTRAP or API 4000; AB SCIEX, Framingham, MA, USA).

Analyte concentrations were determined using calibration curves with terfenadine as the internal standard. Chromatographic data acquisition and peak integration were performed using Analyst software (v1.6.2; AB SCIEX), and peak-area ratios were processed using Watson LIMS™ software (v7.5; Thermo Fisher Scientific, Waltham, MA, USA).

### 2.9. Normal Tissue Biodistribution Analysis

Fifteen female Sprague–Dawley (SD, 6–8 weeks old, 180–200 g) rats were randomly assigned to five terminal sampling cohorts (*n* = 3 per time point). FMM-66 was administered intravenously at a dose of 2.5 mg/kg. Tissue samples were collected at 1, 6, 24, 96, and 168 h after administration. At each predetermined time point, animals were euthanized by cervical dislocation, and major normal tissues, including ovary, brain, breast, stomach, heart, intestine, kidney, liver, lung, and spleen, were collected for quantitative analysis. Sample preparation and LC–MS/MS quantification were performed according to the procedures described in the pharmacokinetic analysis.

### 2.10. In Vivo Safety Evaluation

Fifteen female SD (6–8 weeks old, 180–200 g) rats were enrolled in the safety evaluation study. Animals received intravenous administration of vehicle or FMM-66 at doses of 1.25 and 2.5 mg/kg. Blood samples were collected on days 2 and 14 post-administration. Prior to blood collection, animals were fasted for at least 12 h with free access to water. Approximately 0.9 mL of blood was collected into sodium citrate-containing tubes for plasma preparation and centrifuged at approximately 1800× *g* for 10 min at 15–25 °C. Plasma biochemical parameters, including aspartate aminotransferase (AST), alanine aminotransferase (ALT), creatinine (CREA), and urea (UREA), were measured to assess potential systemic toxicity. Animals were monitored for body-weight changes throughout the 14-day observation period.

### 2.11. Statistical Analysis

Data are presented as mean ± standard error of the mean (SEM). Statistical analyses were performed using GraphPad Prism software (version 7.0; GraphPad Software, La Jolla, CA, USA). EC_50_ and IC_50_ values were determined by nonlinear regression analysis using a three-parameter logistic model. Comparisons between two groups were performed using an unpaired two-tailed Student’s *t*-test, whereas comparisons among multiple groups were conducted using one-way or two-way analysis of variance (ANOVA) followed by Tukey’s multiple comparisons test. Tumor growth curves were analyzed using two-way repeated-measures ANOVA, followed by Dunnett’s multiple-comparisons. All comparisons are reported with 95% confidence intervals. *p* < 0.05 was considered statistically significant, with *p* < 0.05 marked as *, *p* < 0.01 as **, and *p* < 0.001 as ***.

## 3. Results

### 3.1. Design and Synthesis of the Dual-Targeting PDC FMM-66

**Figure 1:**
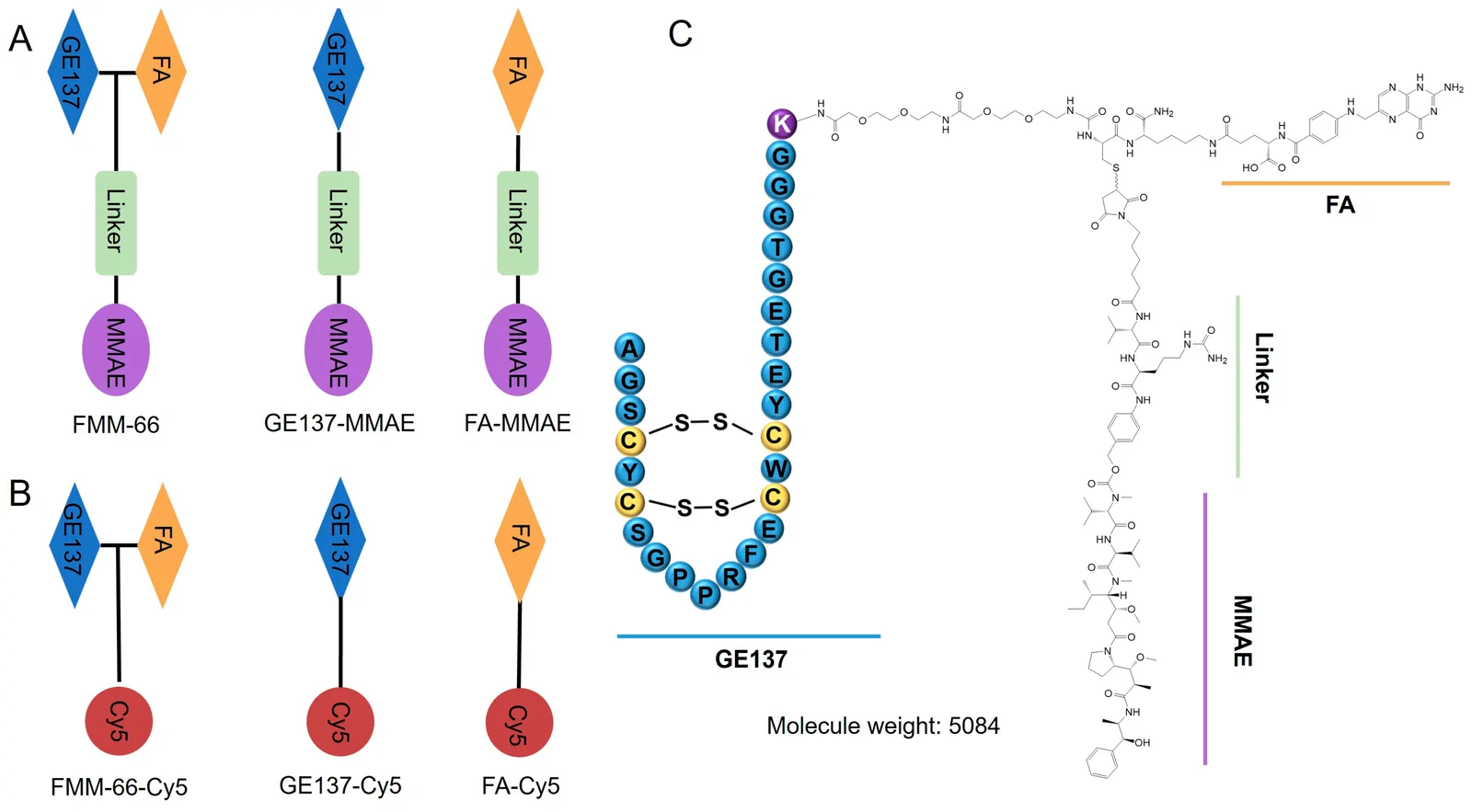
Design and chemical structure of the dual-targeting peptide-drug conjugate FMM-66. (**A**) Schematic illustration of the dual-targeting peptide-drug conjugate FMM-66, consisting of the FOLR1-targeting ligand folic acid (FA) and the c-Met-targeting bicyclic peptide GE137, which are linked through a branched scaffold and conjugated to the cytotoxic payload monomethyl auristatin E (MMAE) via a cathepsin B-cleavable valine-citrulline (Val-Cit) linker. Single-targeting conjugates (GE137-MMAE and FA-MMAE) were synthesized as controls. (**B**) Schematic structures of the corresponding Cy5-labeled probes (FMM-66-Cy5, GE137-Cy5, and FA-Cy5) used for receptor-binding and cellular internalization studies. (**C**) Amino acid sequence of the c-Met-targeting bicyclic peptide GE137 and the chemical structure of FMM-66, highlighting the FOLR1-targeting ligand (FA), enzyme-cleavable linker, and MMAE payload.

Folate receptor alpha (FOLR1) and the mesenchymal–epithelial transition factor (c-Met) are highly expressed in epithelial ovarian cancer (EOC) and represent two clinically validated therapeutic targets. To simultaneously target both receptors, we designed a novel dual-targeting peptide-drug conjugate (PDC), termed FMM-66, by integrating folic acid (FA), a high-affinity ligand for FOLR1, with GE137, a bicyclic peptide that specifically recognizes c-Met. As illustrated in Figure 1A, FA and GE137 were covalently linked through a branched scaffold to generate a dual-ligand targeting moiety. The branched scaffold is a multifunctional covalent conjugate that integrates a GE-137, FA, and the cytotoxic payload monomethyl auristatin E (MMAE) via modular linkers including flexible PEG spacers, amide bonds, maleimide-thioether linkages, a cathepsin-B-cleavable Val-Cit dipeptide, and a para-aminobenzyl (PAB) self-immolative spacer. PEG segments confer high aqueous solubility to counteract MMAE-driven hydrophobic aggregation; the conjugate is soluble in aqueous buffers and polar organic cosolvents but insoluble in non-polar solvents, with a net negative charge at physiological pH 7.4. The Val-Cit dipeptide is designed to be cleaved by lysosomal proteases, including cathepsin B, thereby triggering PAB-mediated self-immolation and release of free MMAE.

To facilitate in vitro characterization, the corresponding Cy5-labeled probes (FMM-66-Cy5, GE137-Cy5, and FA-Cy5) were synthesized by replacing MMAE with the fluorescent dye Cy5 (Figure 1B). The amino acid sequence of GE137 and the chemical structure of FMM-66 are shown in Figure 1C, confirming the successful assembly of the dual-targeting PDC comprising the FOLR1 ligand, the c-Met-targeting peptide, the enzyme-cleavable linker, and the MMAE payload.

### 3.2. FMM-66 Exhibits High-Affinity and Dual-Target-Specific Binding to FOLR1/c-Met-Positive Ovarian Cancer Cells

**Figure 2:**
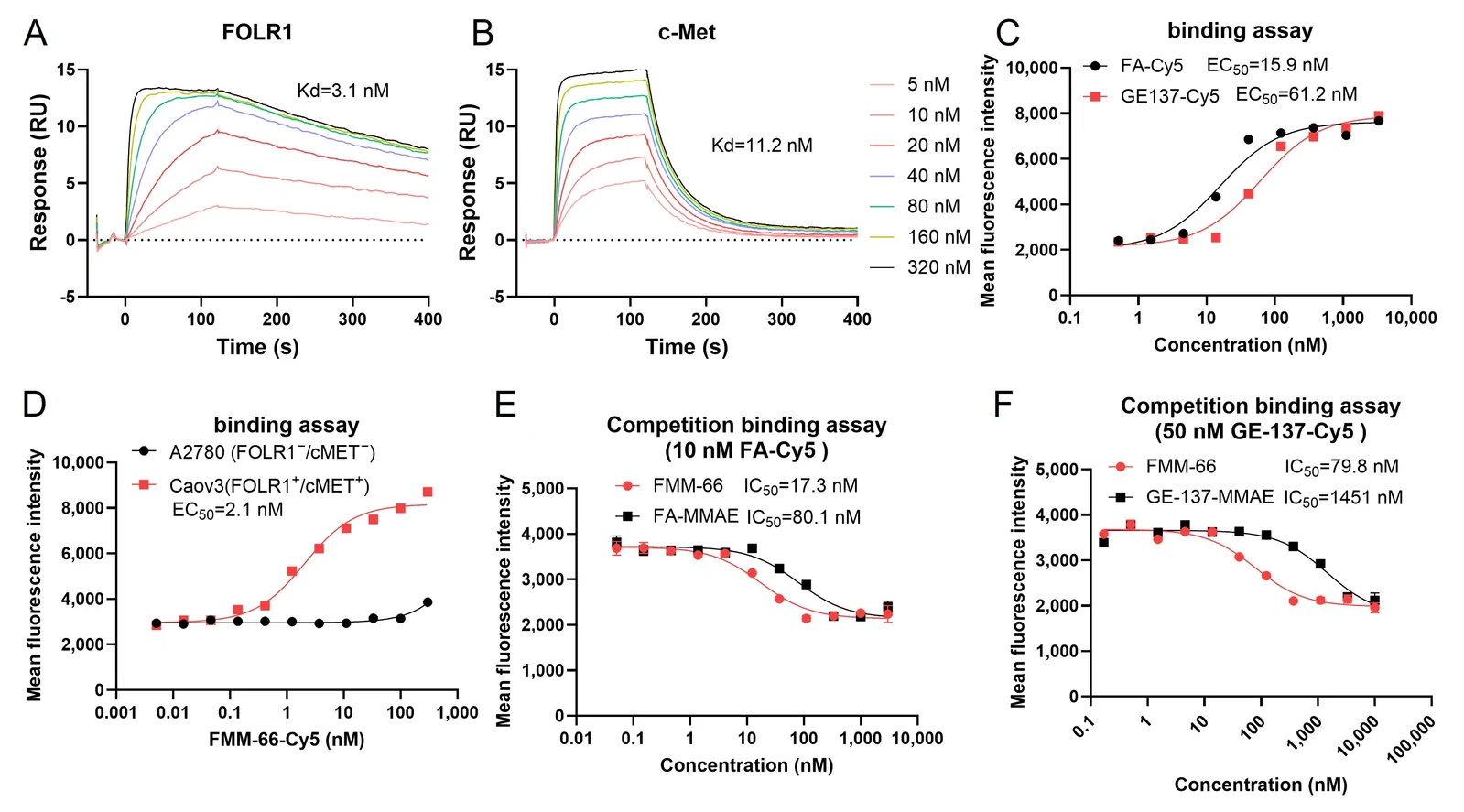
FMM-66 exhibits high-affinity and dual-target-specific binding to FOLR1 and c-Met. (**A**,**B**) Surface plasmon resonance (SPR) sensorgrams showing the binding kinetics of FMM-66 to recombinant human FOLR1 (**A**) and c-Met (**B**). (**C**) Cell-binding saturation assays of FA-Cy5 and GE137-Cy5 in FOLR1/c-Met double-positive Caov3 cells. (**D**) Cell-binding assay of FMM-66-Cy5 in FOLR1/c-Met double-positive Caov3 cells and FOLR1/c-Met double-negative A2780 cells. (**E**) Competitive binding assay for FOLR1 in Caov3 cells using 10 nM FA-Cy5 as the fluorescent tracer. (**F**) Competitive binding assay for c-Met in Caov3 cells using 50 nM GE137-Cy5 as the fluorescent tracer. Data are presented as mean ± SEM (*n* = 3 independent biological experiments). EC_50_ and IC_50_ values were determined by nonlinear regression using a three-parameter logistic model.

To characterize the binding properties of FMM-66, surface plasmon resonance (SPR) analysis was first performed using recombinant human FOLR1 and c-Met proteins. As shown in Figure 2A,B, FMM-66 bound both FOLR1 and c-Met with high affinity, exhibiting equilibrium dissociation constants (Kd) of 3.1 nM and 11.2 nM, respectively, confirming that conjugation of the two targeting ligands did not compromise their receptor-binding capability.

To evaluate receptor binding at the cellular level, Cy5-labeled ligands were incubated with FOLR1/c-Met double-positive Caov3 cells. As shown in Figure 2C, both FA-Cy5 and GE137-Cy5 displayed concentration-dependent binding with EC_50_ values of 15.9 nM and 61.2 nM, respectively, indicating that both receptors are readily accessible on the cell surface. In contrast, the dual-targeting probe FMM-66-Cy5 exhibited markedly enhanced binding to Caov3 cells, with an EC_50_ of 2.1 nM, representing approximately 7.6-fold and 29.1-fold higher binding affinity than FA-Cy5 and GE137-Cy5, respectively (Figure 2D). Importantly, FMM-66-Cy5 showed negligible binding to FOLR1/c-Met double-negative A2780 cells, demonstrating excellent receptor specificity and minimal nonspecific interactions.

The contribution of each targeting ligand to receptor recognition was further investigated by competitive binding assays. In the FOLR1 competition assay, FMM-66 displaced FA-Cy5 binding more efficiently than the single-target conjugate FA-MMAE, yielding IC_50_ values of 17.3 nM and 80.1 nM, respectively (Figure 2E). Likewise, in the c-Met competition assay, FMM-66 exhibited substantially stronger competitive binding than GE137-MMAE, with IC_50_ values of 79.8 nM versus 1451 nM (Figure 2F). To further determine the contribution of each receptor to FMM-66 binding, receptor-blocking experiments were performed in Caov3 cells. The EC_50_ of FMM-66-Cy5 increased from 2.5 nM under vehicle conditions to 97 nM following FOLR1 blockade with excess folate and to 18 nM following c-Met blockade with an anti-c-Met antibody. Simultaneous blockade of both receptors markedly reduced FMM-66-Cy5 binding, with the EC_50_ increasing to >10,000 nM (Figure S8). These results indicate that both FOLR1 and c-Met contribute to the cellular binding of FMM-66.

Flow-cytometric analysis was further performed to quantitatively characterize FOLR1 and c-Met expression across the ovarian cancer cell lines used in this study. Caov3 and SKOV3 exhibited relatively high expression of both FOLR1 and c-Met, whereas JHOS4 predominantly expressed FOLR1 and DOV13 predominantly expressed c-Met. In contrast, A2780 and COV434 showed relatively low expression of both receptors (Figure S9). Collectively, these findings demonstrate that FMM-66 retains binding capability toward both FOLR1 and c-Met and that both receptors contribute to its enhanced cellular binding in dual-receptor-expressing Caov3 cells.

### 3.3. FOLR1-Mediated Internalization Dominates the Cellular Uptake of FMM-66

**Figure 3:**
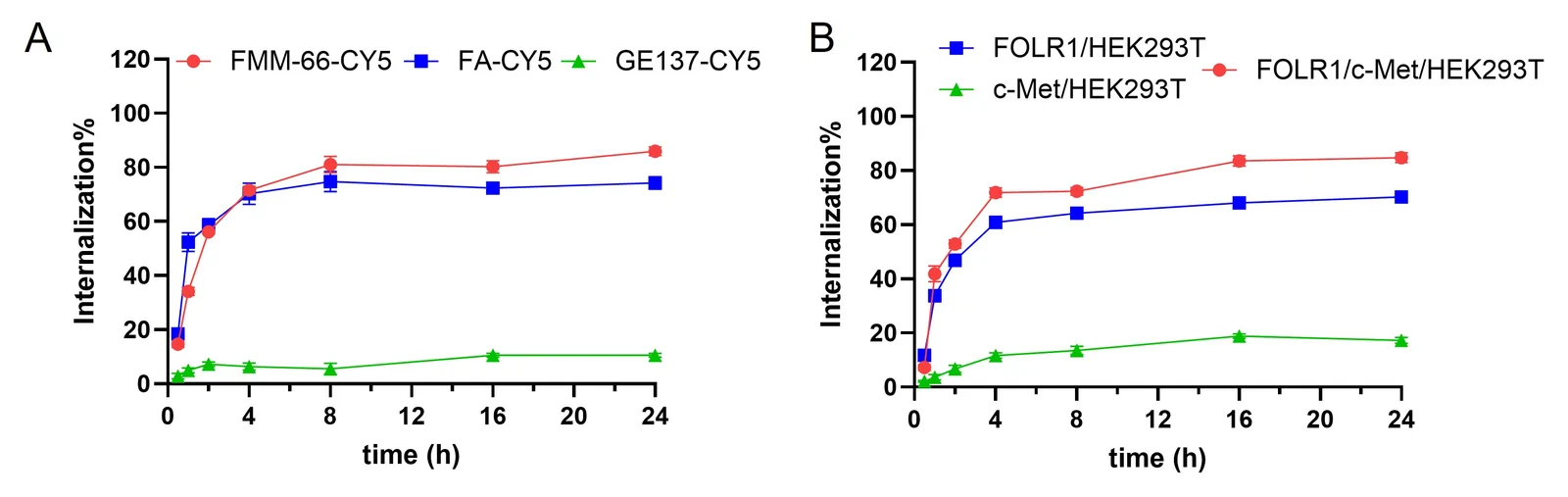
FOLR1-mediated internalization dominates the cellular uptake of FMM-66. (**A**) Internalization kinetics of FMM-66-Cy5, FA-Cy5, and GE137-Cy5 in FOLR1/c-Met double-positive Caov3 cells. (**B**) Internalization of FMM-66-Cy5 in HEK293T cells overexpressing FOLR1, c-Met, or both receptors. Data are presented as mean ± SEM (*n* = 3 independent biological experiments).

Efficient intracellular delivery is essential for the therapeutic activity of peptide-drug conjugates. We therefore compared the internalization kinetics of FMM-66-Cy5 with those of the corresponding single-ligand probes in FOLR1/c-Met double-positive Caov3 cells. As shown in Figure 3A, both FMM-66-Cy5 and FA-Cy5 were rapidly internalized, with approximately 70% of the cell-associated fluorescence becoming acid-resistant within 4 h, and FMM-66-Cy5 reached an internalization efficiency of approximately 85% after 24 h. In contrast, GE137-Cy5 exhibited minimal internalization throughout the experiment, with only ~10% internalized after 24 h, indicating that c-Met binding alone does not efficiently trigger receptor-mediated endocytosis.

To further investigate the respective contributions of FOLR1 and c-Met to FMM-66 internalization, HEK293T cells overexpressing FOLR1, c-Met, or both receptors were established. As shown in Figure 3B, FMM-66 was efficiently internalized in FOLR1-overexpressing cells, reaching approximately 70% internalization at 24 h, whereas only ~17% internalization was observed in cells expressing c-Met alone. Notably, dual overexpression of FOLR1 and c-Met further increased FMM-66 internalization to approximately 85% at 24 h, consistent with the results obtained in Caov3 cells.

Collectively, these findings indicate that FOLR1-mediated uptake is the predominant internalization route for FMM-66, whereas c-Met alone supports only limited internalization. The higher internalization observed in dual-receptor-overexpressing cells suggests that c-Met engagement may provide an additional contribution in the presence of FOLR1.

### 3.4. Cathepsin B-Mediated Cleavage of FMM-66 Results in Time-Dependent MMAE Release

Following receptor-mediated internalization, FMM-66 is expected to traffic to lysosomes, where the valine-citrulline (Val-Cit) linker is cleaved by lysosomal proteases to release the cytotoxic payload MMAE. To evaluate the enzymatic stability and payload release profile of FMM-66, the conjugate was incubated with recombinant human cathepsin B, the principal protease responsible for Val-Cit linker cleavage [28].

**Figure 4:**
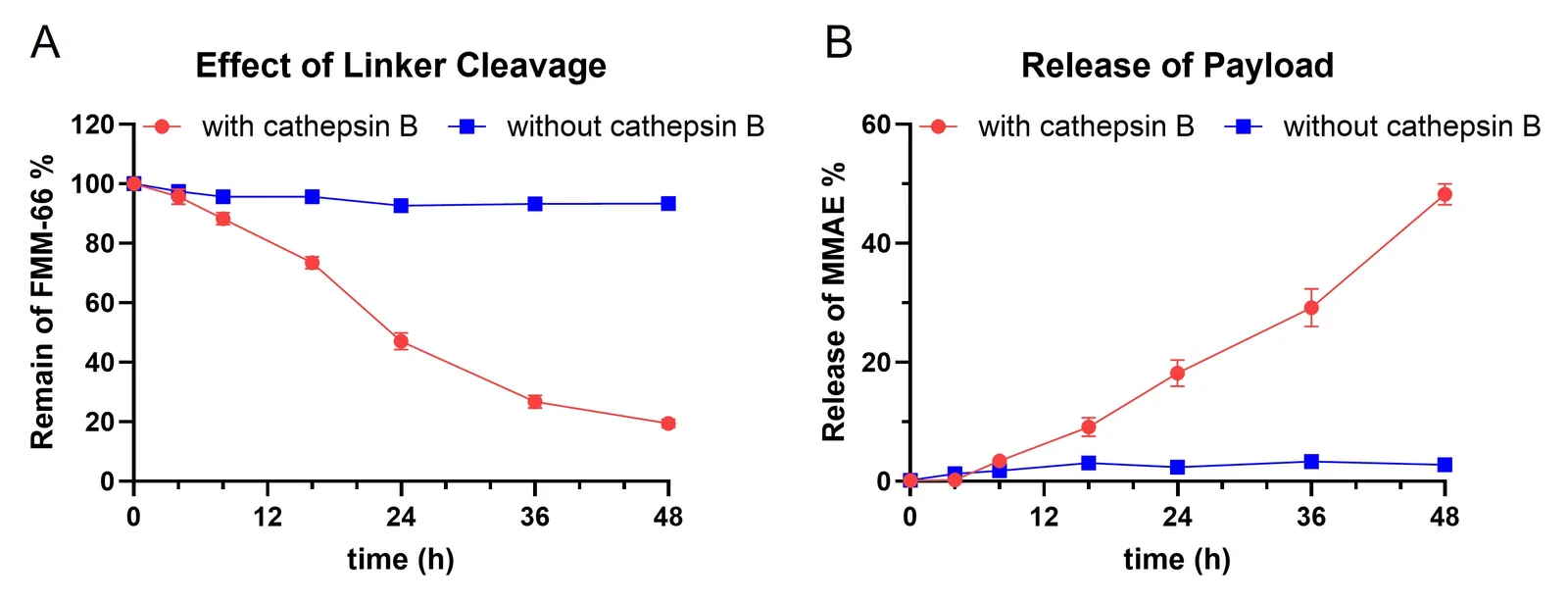
Cathepsin B-mediated cleavage of the Val-Cit linker and release of MMAE from FMM-66. (**A**) Time-dependent degradation of intact FMM-66 following incubation with recombinant human cathepsin B or buffer control. The percentage of intact FMM-66 was quantified by LC–MS/MS at the indicated time points. (**B**) Time-dependent release of MMAE from FMM-66 in the presence or absence of cathepsin B. Released MMAE was quantified by LC–MS/MS. Data are presented as mean ± SEM (*n* = 3 independent biological experiments).

As shown in Figure 4A, FMM-66 underwent progressive enzymatic degradation in the presence of cathepsin B. The percentage of intact FMM-66 decreased from 100% at baseline to 96%, 88%, 73%, 47%, 27%, and 19% after 4, 8, 16, 24, 36, and 48 h, respectively. In contrast, FMM-66 remained highly stable in the absence of cathepsin B, with 93% of the intact conjugate still detected after 48 h, indicating excellent linker stability under non-enzymatic conditions.

Consistent with linker cleavage, MMAE was released in a time-dependent manner following incubation with cathepsin B (Figure 4B). The cumulative release of MMAE reached 0.1%, 0.2%, 3.4%, 9.1%, 18.2%, 29.2%, and 48.2% at 0, 4, 8, 16, 24, 36, and 48 h, respectively, whereas negligible MMAE release (<3%) was detected in the enzyme-free control throughout the incubation period. Notably, the extent of intact FMM-66 loss exceeded the amount of free MMAE detected, likely because cathepsin B-mediated cleavage generated linker- and/or pay-load-containing intermediates that were not quantified by the free-MMAE assay. To further assess the functional relevance of cathepsin B-dependent processing, we examined the effect of CA-074 [29], a potent cathepsin B inhibitor, on FMM-66-mediated cytotoxi-city. CA-074 (5 nM) alone had no appreciable effect on Caov3 cell viability, whereas co-treatment markedly attenuated FMM-66-mediated cytotoxicity, shifting the IC50 from 8.3 nM to >10,000 nM (Figure S10). Collectively, these results demonstrate that FMM-66 undergoes cathepsin B-mediated cleavage accompanied by time-dependent MMAE release. Furthermore, the marked attenuation of FMM-66 cytotoxicity by CA-074 provides functional evidence that cathepsin B-dependent processing contributes substantially to FMM-66 activation and subsequent cytotoxicity in Caov3 cells.

### 3.5. FMM-66 Exhibits Potent and Target-Dependent Cytotoxicity in Ovarian Cancer Cells

**Figure 5:**
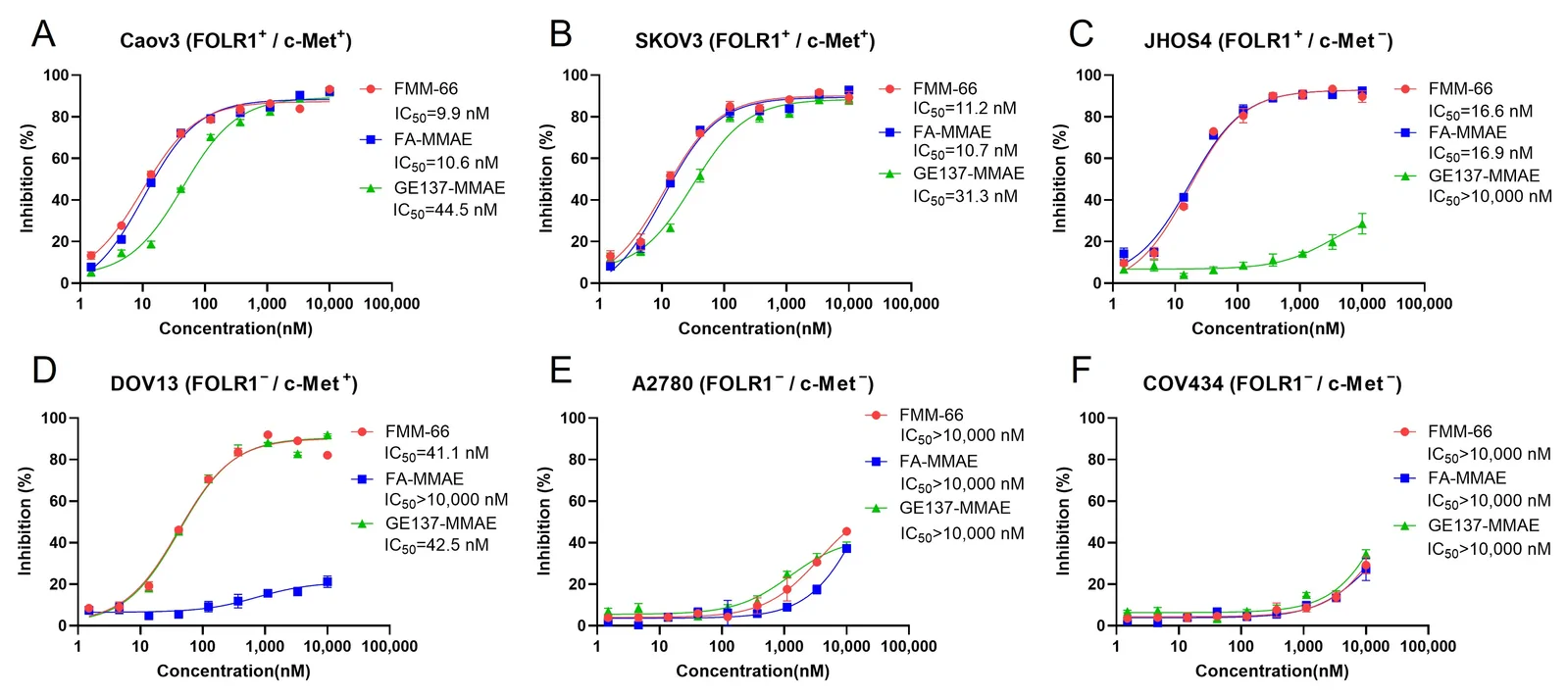
Target-dependent cytotoxic activity of FMM-66 in ovarian cancer cell lines. (**A**,**B**) Cytotoxic effects of FMM-66, FA-MMAE, and GE137-MMAE in FOLR1/c-Met double-positive Caov3 (**A**) and SKOV3 (**B**) cells after 72 h of treatment. (**C**) Cytotoxicity in FOLR1-positive/c-Met-negative JHOS4 cells. (**D**) Cytotoxicity in FOLR1-negative/c-Met-positive DOV13 cells. (**E**,**F**) Cytotoxicity in FOLR1/c-Met double-negative A2780 (**E**) and COV434 (**F**) cells. Cell viability was determined using the CellTiter-Glo assay after 72 h of incubation. Data are presented as mean ± SEM (*n* = 3 independent biological experiments). IC_50_ values were calculated by nonlinear regression using a three-parameter logistic model.

To evaluate the antitumor activity of FMM-66, cell viability was assessed in ovarian cancer cell lines with different FOLR1 and c-Met expression profiles following 72 h of treatment. As shown in Figure 5A and Figure 5B, FMM-66 exhibited potent cytotoxic activity in FOLR1/c-Met double-positive Caov3 and SKOV3 cells, with IC_50_ values of 9.9 nM and 11.2 nM, respectively. These activities were comparable to those of the FOLR1-targeting conjugate FA-MMAE (IC_50_ = 10.6 and 10.7 nM) and markedly superior to those of the c-Met-targeting conjugate GE137-MMAE (IC_50_ = 44.5 and 31.3 nM), consistent with the rapid FOLR1-mediated internalization observed previously.

The target dependence of FMM-66 was further investigated using receptor-differential cell lines. In JHOS4 cells, which express high FOLR1 but low c-Met (Figure S9, Figure 5C), FMM-66 retained potent cytotoxicity with an IC_50_ of 16.6 nM, comparable to FA-MMAE (IC_50_ = 16.9 nM), whereas GE137-MMAE exhibited minimal activity (IC_50_ > 1000 nM). Conversely, in DOV13 cells, which express high c-Met but low FOLR1 (Figure S9, Figure 5D), FMM-66 displayed cytotoxicity comparable to GE137-MMAE, with IC_50_ values of 41.1 nM and 42.5 nM, respectively, while FA-MMAE showed negligible activity (IC_50_ > 10,000 nM). The measurable activity of GE137-MMAE in DOV13 despite its relatively low internalization efficiency likely reflects the high c-Met surface abundance in this cell line together with prolonged 72-h exposure to the highly potent MMAE payload; nevertheless, its potency remained lower than that of FOLR1-directed conjugates in FOLR1-high cells.

To further assess target specificity, cytotoxicity was examined in FOLR1/c-Met double-negative A2780 and COV434 cells. As shown in Figure 5E,F, all three conjugates exhibited minimal antiproliferative activity, with IC_50_ values exceeding 10,000 nM, indicating negligible nonspecific toxicity in the absence of both target receptors.

Collectively, these results demonstrate that the cytotoxic activity of FMM-66 is highly dependent on target receptor expression. Notably, FMM-66 and FA-MMAE exhibited comparable in vitro cytotoxic potency in FOLR1-high cells, whereas FMM-66 retained cytotoxic activity in c-Met-high/FOLR1-low DOV13 cells.

### 3.6. Dual-Targeting Enhances Tumor Accumulation and Improves the In Vivo Antitumor Efficacy of FMM-66

**Figure 6:**
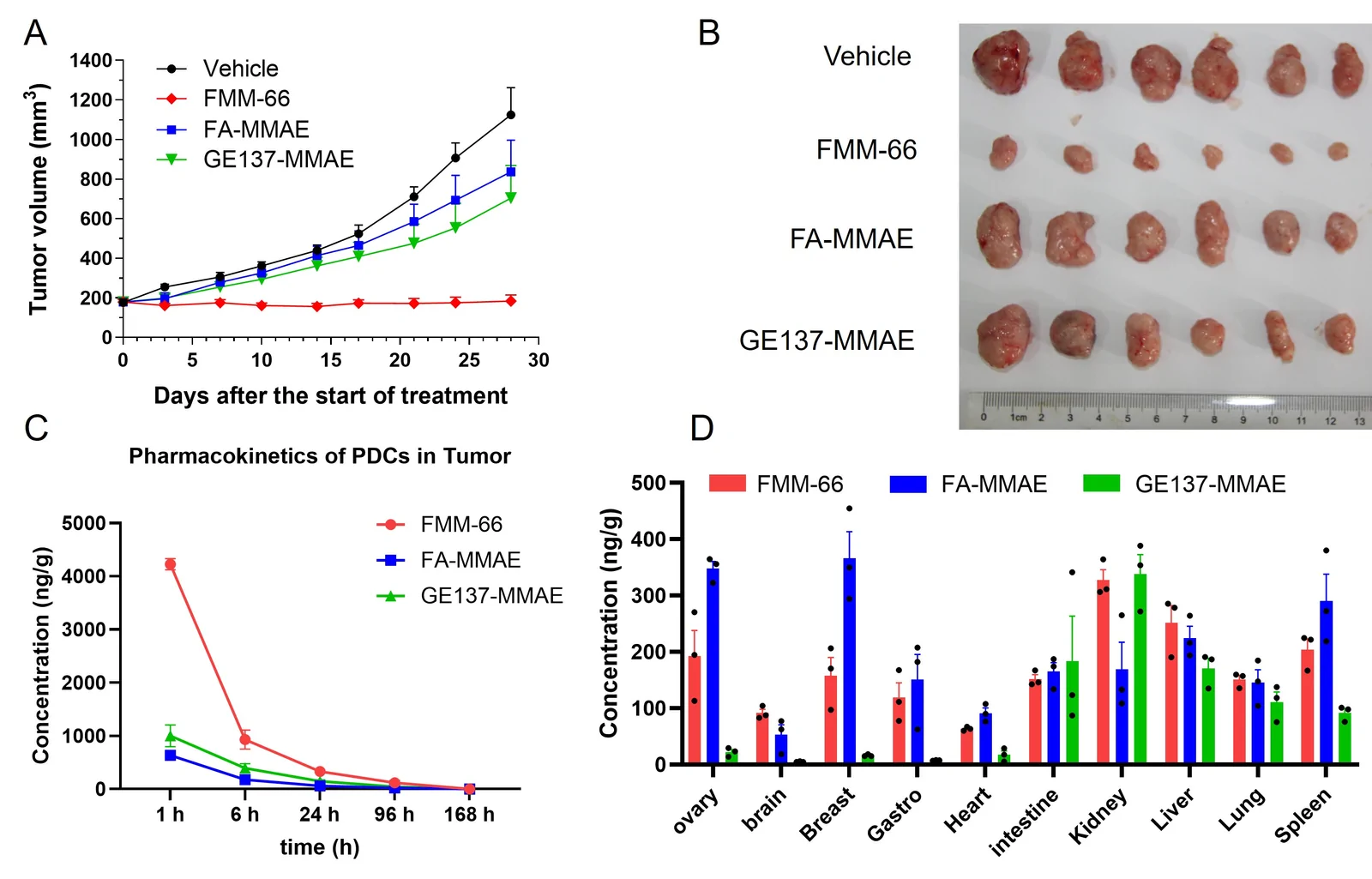
Dual-targeting improves tumor accumulation and enhances the antitumor efficacy of FMM-66 in vivo. (**A**) Antitumor efficacy of FMM-66, FA-MMAE, and GE137-MMAE in a Caov3 cell-derived xenograft (CDX) model. Treatment was initiated when tumor volumes reached approximately 200 mm^3^. Mice (*n* = 6 biologically independent animals per group) received intravenous administration of equimolar doses once weekly for three consecutive weeks (QW × 3), and tumor volumes were measured twice weekly. (**B**) Representative images of excised tumors collected at the end of the study. (**C**) Intratumoral pharmacokinetic profiles of FMM-66, FA-MMAE, and GE137-MMAE following a single intravenous administration. Drug concentrations in tumor tissues were quantified by LC–MS/MS at the indicated time points (*n* = 3 biologically independent animals). (**D**) Tissue distribution of FMM-66, FA-MMAE, and GE137-MMAE in major organs 24 h after intravenous administration. Drug concentrations were determined by LC–MS/MS (*n* = 3 biologically independent animals). Data are presented as mean ± SEM.

To evaluate the in vivo antitumor efficacy of FMM-66, a Caov3 cell-derived xenograft (CDX) model was established in BALB/c nude mice. When tumors reached approximately 200 mm^3^, mice were randomized into treatment groups (n = 6 per group) and intravenously administered equimolar doses (0.5 μmol/kg) of FMM-66 (2.5 mg/kg), FA-MMAE (1.0 mg/kg), or GE137-MMAE (2.25 mg/kg) once weekly for three consecutive weeks (QW × 3). Tumor volumes were monitored twice weekly throughout the study. As shown in Figure 6A, FMM-66 markedly suppressed tumor growth compared with both single-target conjugates. At the study endpoint, FMM-66 achieved a tumor growth inhibition (TGI) of >85%, whereas FA-MMAE and GE137-MMAE produced TGIs of only 25.6% and 38.4%, respectively. Representative excised tumors further confirmed the superior antitumor activity of FMM-66 (Figure 6B). No apparent clinical abnormalities or significant body weight loss were observed in any treatment group during the study, indicating good in vivo tolerability.

Although FA-MMAE exhibited potent receptor binding, rapid internalization, and strong cytotoxicity in vitro, its therapeutic efficacy in vivo was substantially lower than that of FMM-66. To investigate this discrepancy, the intratumoral pharmacokinetics of the three PDCs were determined following a single intravenous administration. As shown in Figure 6C, FMM-66 achieved rapid and sustained accumulation within tumor tissue, reaching 4227 ng/g at 1 h, followed by concentrations of 930 ng/g, 331 ng/g, and 118 ng/g at 6, 24, and 96 h, respectively. In contrast, FA-MMAE exhibited markedly lower intratumoral exposure throughout the study, indicating insufficient tumor accumulation despite its favorable in vitro activity.

To further evaluate tissue distribution, drug concentrations in major organs were quantified 24 h after administration (Figure 6D). FMM-66 showed lower concentrations than FA-MMAE in several normal tissues, including the ovary, breast, stomach, and spleen, while maintaining substantially greater tumor exposure. These findings demonstrate a distinct biodistribution profile of FMM-66 characterized by enhanced tumor accumulation and reduced exposure in several normal tissues relative to FA-MMAE.

### 3.7. FMM-66 Exhibits Rapid Systemic Clearance While Maintaining Prolonged Intratumoral Exposure

**Figure 7:**
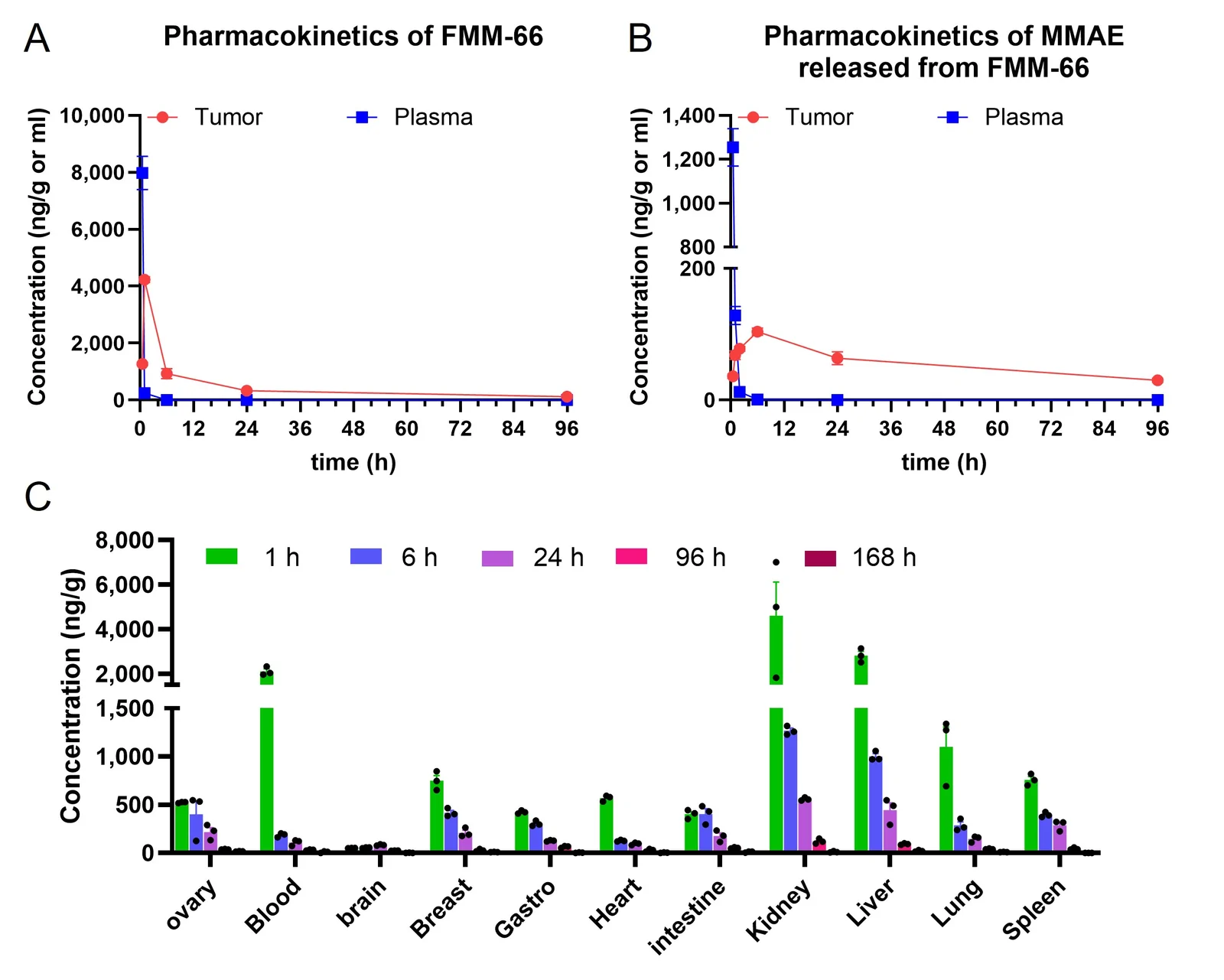
Pharmacokinetic profile and tissue distribution of FMM-66 following intravenous administration. (**A**,**B**) Pharmacokinetic profiles of intact FMM-66 (**A**) and the released MMAE (**B**) in plasma and tumor tissues following a single intravenous administration of FMM-66 (2.5 mg/kg) in Caov3 tumor-bearing mice. Concentrations of intact FMM-66 and MMAE were quantified by LC–MS/MS at the indicated time points. (**C**) Tissue distribution of intact FMM-66 in female Sprague–Dawley rats following a single intravenous administration of 2.5 mg/kg. Drug concentrations in major organs were determined by LC–MS/MS at 1, 6, 24, 96, and 168 h after dosing. Data are presented as mean ± SEM (*n* = 3 biologically independent animals).

To characterize the pharmacokinetic behavior of FMM-66, Caov3 tumor-bearing mice received a single intravenous administration of 2.5 mg/kg FMM-66. The concentrations of intact FMM-66 and its released payload MMAE were quantified in plasma and tumor tissues by LC–MS/MS. As shown in Figure 7A, plasma concentrations of intact FMM-66 declined rapidly after administration and became nearly undetectable within 6 h, whereas tumor concentrations remained substantially higher throughout the observation period. Similarly, the released payload MMAE was rapidly eliminated from the circulation but accumulated within tumor tissues (Figure 7B). Plasma MMAE was almost completely cleared by 6 h, while intratumoral MMAE concentrations gradually increased and remained detectable for at least 24 h. The maximal intratumoral MMAE concentration (Cmax) reached 103 ng/g, with an AUC(0–t) of 4291 h*ng/g, demonstrating sustained intracellular payload exposure within tumors despite rapid systemic clearance. Corresponding pharmacokinetic parameters are provided in Table S1: the terminal half-lives of intact FMM-66 were 0.75 h in plasma and 4.1 h in tumor, whereas released MMAE showed terminal half-lives of 0.78 h in plasma and 48.6 h in tumor.

To further evaluate the tissue distribution of FMM-66, female Sprague–Dawley rats received a single intravenous dose of 2.5 mg/kg, and tissue concentrations were determined at multiple time points. As shown in Figure 7C, FMM-66 reached peak concentrations in most tissues at 1 h after administration and subsequently declined rapidly. The highest initial drug exposure was observed in the kidney, followed by the liver, lung, spleen, breast, ovary, intestine, heart, and brain, consistent with the physiological roles of the kidney and liver in drug elimination. At 24 h, relatively higher concentrations remained only in the kidney (562 ng/g) and liver (493 ng/g), whereas drug levels in other tissues had markedly decreased. By 96 h, FMM-66 concentrations had declined substantially in all tissues, and only trace amounts remained at 168 h, indicating efficient systemic elimination.

Collectively, these findings demonstrate that FMM-66 combines rapid clearance from the circulation with sustained intratumoral retention of the released MMAE, while exhibiting efficient elimination from normal tissues. This pharmacokinetic profile is consistent with enhanced tumor exposure while limiting prolonged systemic exposure; however, the high early kidney and liver concentrations warrant consideration in safety assessment.

### 3.8. FMM-66 Exhibits Favorable Systemic Safety with Reversible Hepatic and Renal Toxicity

To evaluate the systemic safety of FMM-66, a single-dose toxicity study was performed in female Sprague–Dawley rats. Animals received a single intravenous administration of vehicle or FMM-66 at 1.25 mg/kg (therapeutic-equivalent dose) or 2.5 mg/kg (high-dose toxicity group). Plasma biochemical parameters were determined on Days 2 and 14 after administration.

**Figure 8:**
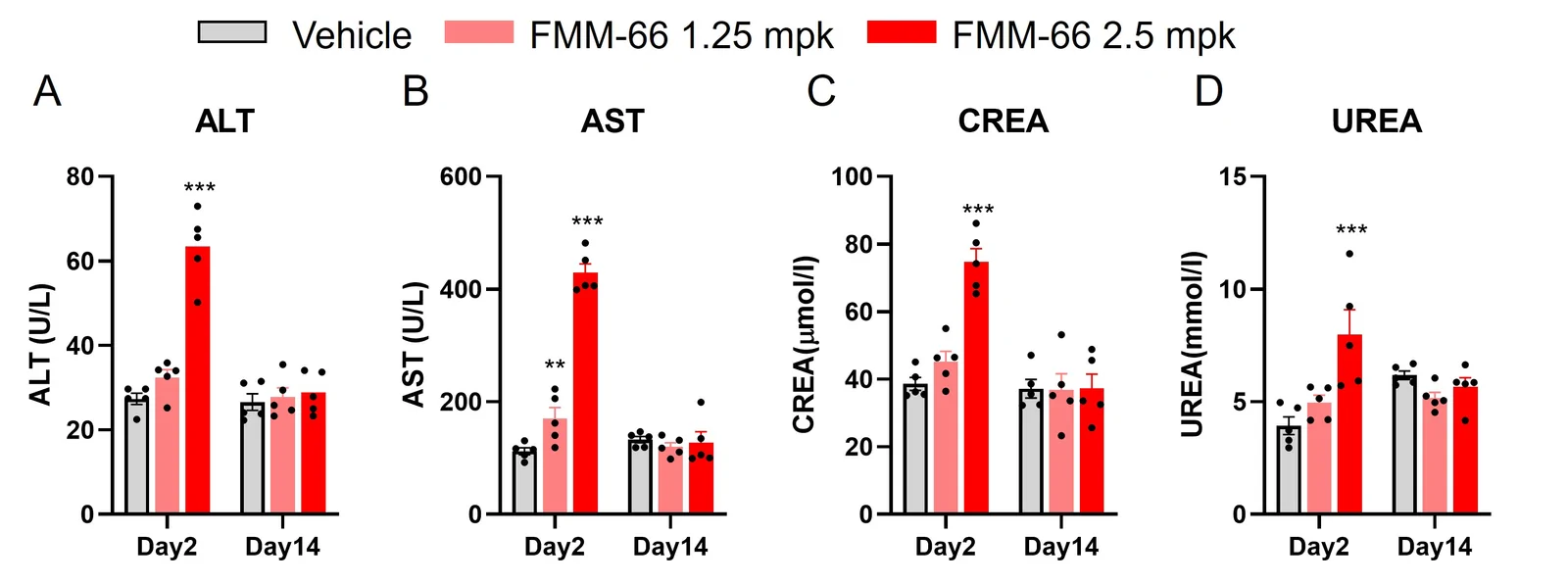
Systemic safety evaluation of FMM-66 following single-dose administration in female Sprague–Dawley rats. Plasma biochemical parameters were measured on Days 2 and 14 after a single intravenous administration of vehicle or FMM-66 at 1.25 mg/kg or 2.5 mg/kg. (**A**) Alanine aminotransferase (ALT). (**B**) Aspartate aminotransferase (AST). (**C**) Creatinine (CREA). (**D**) Urea (UREA). Data are presented as mean ± SEM (*n* = 5 biologically independent animals per group) and were analyzed by two-way ANOVA with multiple comparisons test. ** *p* < 0.01, *** *p* < 0.001 vs. vehicle.

As shown in Figure 8, administration of 1.25 mg/kg FMM-66 produced no significant changes in serum biochemical parameters compared with the vehicle group. In contrast, rats receiving 2.5 mg/kg FMM-66 exhibited transient elevations in liver and kidney function biomarkers on Day 2, including alanine aminotransferase (ALT), aspartate aminotransferase (AST), creatinine (CREA), and urea (UREA), suggesting temporary hepatic and renal stress following high-dose administration.

By Day 14, ALT, AST, CREA, and UREA values had largely returned to levels comparable to those of vehicle-treated animals. In addition, no treatment-related body-weight loss was observed at either dose during the 14-day observation period (Figure S11). Collectively, these findings indicate that FMM-66 was generally well tolerated at 1.25 mg/kg under the conditions of this single-dose study, while the biochemical alterations observed at 2.5 mg/kg were transient and largely resolved by Day 14. These results provide preliminary evidence of tolerability of FMM-66 at the tested dose levels.

## 4. Discussion

ADCs and PDCs have transformed precision oncology by enabling selective delivery of highly potent cytotoxic payloads to tumor cells [30,31]. Nevertheless, their therapeutic efficacy is frequently constrained by the “on-target, off-tumor” effect, a fundamental pharmacological limitation arising from physiological expression of target antigens in normal tissues. Although FOLR1 is highly expressed in the majority of epithelial ovarian cancers, it is also present in several normal epithelial tissues, including the ovary, breast, kidney, and gastrointestinal tract. Consequently, single-target FOLR1-directed conjugates can bind both tumor and normal tissues after systemic administration, resulting in drug sequestration, reduced tumor exposure, and dose-limiting toxicity. Improving tumor selectivity while maintaining efficient intracellular drug delivery therefore remains a major challenge for next-generation targeted therapeutics.

In the present study, we addressed this challenge by developing FMM-66, a dual-ligand peptide-drug conjugate simultaneously targeting FOLR1 and c-Met. Unlike conventional dual-target strategies that primarily aim to increase binding affinity, the rationale of FMM-66 was to exploit the differential co-expression pattern of FOLR1 and c-Met between tumors and normal tissues. While both receptors are highly co-expressed in ovarian cancer, simultaneous high-level expression is uncommon in normal organs. This dual-recognition requirement therefore increases the probability of selective tumor binding while reducing interaction with normal tissues expressing only one receptor.

Our results consistently support this design concept. SPR analysis confirmed that FMM-66 retained high-affinity binding toward both FOLR1 and c-Met, while cellular binding assays demonstrated substantially enhanced receptor binding compared with either single-ligand conjugate. Importantly, receptor blockade of either FOLR1 or c-Met reduced FMM-66-Cy5 binding, whereas combined blockade nearly abolished it, demonstrating contributions from both ligands. Biodistribution studies further revealed markedly greater tumor accumulation of FMM-66 than FA-MMAE, despite the comparable in vitro binding, internalization, and cytotoxicity of the two constructs. This observation suggests that the superior in vivo efficacy of FMM-66 is not simply attributable to increased receptor affinity but rather to improved tumor-selective distribution. In contrast, FA-MMAE preferentially accumulated in multiple normal organs expressing physiological levels of FOLR1, including the ovary, breast, stomach, and spleen, thereby limiting its availability for tumor uptake. The lower normal-tissue exposure of FMM-66 relative to FA-MMAE should not be attributed solely to dual-receptor recognition, because FMM-66 can still bind FOLR1-positive tissues. Differences in molecular size, physicochemical properties, tissue permeability, receptor density, and clearance may also contribute.

Another important finding of this study concerns the distinct biological roles of the two targeting ligands. Although c-Met represents an attractive therapeutic target because of its high expression in multiple malignancies and relatively restricted distribution in normal tissues, GE137 alone exhibited minimal receptor-mediated internalization [21]. Consequently, GE137-MMAE showed limited intracellular drug delivery despite maintaining receptor binding. Our mechanistic studies demonstrated that FOLR1 serves as the principal driver of receptor-mediated endocytosis, whereas GE137 primarily contributes to tumor recognition and selective binding. The integration of these complementary functions allows FMM-66 to overcome a common limitation encountered during ligand-drug conjugate development, namely that many high-affinity targeting ligands lack efficient internalization capability and therefore cannot function as effective drug carriers. The dual-ligand architecture effectively “rescues” such ligands by coupling them to an internalizing receptor, thereby broadening the range of targeting ligands that may be suitable for PDC development.

Pharmacokinetic analysis further demonstrated another important advantage of FMM-66. Intact FMM-66 and free MMAE were rapidly cleared from the circulation, whereas released MMAE remained detectable within tumor tissues for prolonged periods. This pharmacokinetic behavior supports the proposed mechanism of action, in which FMM-66 rapidly reaches the tumor through receptor-mediated targeting. This pharmacokinetic behavior is consistent with a proposed mechanism in which FMM-66 accumulates in tumor tissue following receptor-mediated targeting and internalization, followed by proteolytic processing and local release of MMAE. Rapid systemic clearance may further reduce normal tissue exposure to both intact conjugate and released payload, thereby contributing to an improved therapeutic index.

Safety evaluation supported the preliminary tolerability profile of FMM-66. At the therapeutic-equivalent dose (1.25 mg/kg in rats), no significant abnormalities were observed in the serum biochemical parameters examined. Although transient elevations of ALT, AST, CREA, and UREA were observed at the higher dose (2.5 mg/kg), these changes largely returned toward levels observed in the vehicle group by Day 14, suggesting that the observed hepatic and renal biochemical alterations were reversible under the conditions tested. Together with the absence of body weight loss during efficacy studies, these findings indicate that FMM-66 has a certain safety margin at the tested dose levels, although the available data are insufficient to establish a broad therapeutic window. Importantly, the present safety assessment was based on a single-dose acute toxicity study and therefore provides only a preliminary evaluation of the safety profile of FMM-66. Future studies will include repeated-dose subacute toxicity assessments that more closely reflect the therapeutic dosing regimen, together with histopathological examination of major organs and more comprehensive serum biochemical and hematological analyses, to systematically characterize the potential cumulative and organ-specific toxicities of FMM-66 and more accurately define its therapeutic window.

Despite these encouraging findings, several limitations should be acknowledged. First, the cellular binding and internalization assays were performed using Cy5-labeled analogues. Because replacement of MMAE with Cy5 may alter molecular size, charge, hydrophobicity, and membrane interactions, the Cy5-labeled constructs may not fully reproduce the cellular behavior of the corresponding MMAE-containing conjugates. Thus, these data should primarily be interpreted as evidence of receptor-dependent targeting and uptake rather than as a quantitative surrogate for FMM-66 itself. Direct comparison of the physicochemical properties, receptor-binding affinities, and cellular uptake of the Cy5- and MMAE-containing conjugates will be required in future studies. Second, the current study employed a subcutaneous Caov3 cell line-derived xenograft model, which does not fully recapitulate the anatomical site, metastatic behavior, heterogeneity, or stromal complexity of human ovarian cancer. Validation in orthotopic, patient-derived xenograft, or additional models spanning different FOLR1/c-Met expression ratios will be important to determine how receptor abundance influences the benefit of dual targeting. Third, the use of mice for efficacy/tumor pharmacokinetics and rats for normal-tissue distribution and safety was intended to address complementary experimental objectives, but interspecies differences limit direct quantitative comparison across datasets. Fourth, although the recombinant cathepsin B cleavage assay and CA-074 inhibition experiment support a functional contribution of cathepsin B-dependent processing, intracellular release of free MMAE and lysosomal localization of FMM-66 were not directly demonstrated. Future studies combining quantitative intracellular payload analysis with lysosomal colocalization and trafficking assays will be required to further define the intracellular processing mechanism of FMM-66.

## 5. Conclusions

In conclusion, this study provides preclinical proof of concept for FMM-66 as a dual-ligand drug conjugate co-targeting FOLR1 and c-Met. By combining tumor-selective dual-receptor recognition with efficient receptor-mediated internalization and favorable pharmacokinetic behavior, FMM-66 achieves enhanced tumor accumulation and potent antitumor efficacy, and demonstrates preliminary evidence of tolerability at the tested dose levels. These findings provide a framework for further development of multi-ligand drug conjugates designed to improve tumor-selective payload delivery.

## Data Availability

The original contributions presented in this study are included in the article. Further inquiries can be directed to the corresponding authors.

## Supplementary Materials

The following supporting information can be downloaded at: https://www.mdpi.com/article/10.3390/cancers18183013/s1, Figure S1: HPLC purity and LC–MS characterization of FMM-66; Figures S2: HPLC purity and LC–MS characterization of FA-MMAE; Figures S3: HPLC purity and LC–MS characterization of GE137-MMAE; Figures S4: HPLC purity and LC–MS characterization of FMM-66-Cy5; Figures S5: HPLC purity and LC–MS characterization of FA-Cy5; Figures S6: HPLC purity and LC–MS characterization of GE137-Cy5; Figure S7: Chemical structure and molecular weight of GE137-MMAE, FA-MMAE, FA-cy5, FMM-66-Cy5 and GE137-Cy5; Figure S8: Receptor-blocking analysis of FMM-66-Cy5 binding to FOLR1/c-Met co-expressing Caov3 cells.; Figure S9: Expression profiles of c-Met and FOLR1 in ovarian cancer cell lines; Figure S10: Cathepsin B inhibition attenuates FMM-66-mediated cytotoxicity in Caov3 cells; Figure S11: Effect of a single intravenous dose of FMM-66 on body weight in female SD rats; Table S1: Pharmacokinetic profiles of intact FMM-66 and released MMAE in plasma and tumor tissues following a single intravenous administration of FMM-66 (2.5 mg/kg) in Caov3 tumor-bearing mice.

## Abbreviations

The following abbreviations are used in this manuscript:

EOC	Epithelial ovarian cancer
PDC	Peptide-drug conjugate
FOLR1	Folate receptor alpha
GPI	glycosylphosphatidylinositol
ADCs	antibody–drug conjugates
FDA	Food and Drug Administration
c-Met	mesenchymal–epithelial transition factor
HGF	hepatocyte growth factor
FBS	fetal bovine serum
P/S	penicillin/streptomycin
STR	short tandem repeat
SPR	Surface plasmon resonance
BSA	bovine serum albumin
HBSS	Hanks’ balanced salt solution
MFI	mean fluorescence intensity
IACUC	Institutional Animal Care and Use Committee
TGI	Tumor growth inhibition
AST	aspartate aminotransferase
ALT	alanine aminotransferase
CREA	creatinine
SEM	standard error of the mean
FA	folic acid
CDX	cell-derived xenograft
