# Supplementary material for: A Dual-Ligand PDC Co-Targeting FOLR1 and c-Met Enhances Tumor Accumulation and Antitumor Efficacy in Ovarian Cancer
Source: Cancers (Basel). 2026 Sep 17;18(18):3013. doi: 10.3390/cancers18183013 (PMC13604648; doi:10.3390/cancers18183013)
Supplement: Supplementary file 1 [file cancers-18-03013-s001.zip › cancers-4514537-supplementary.pdf]

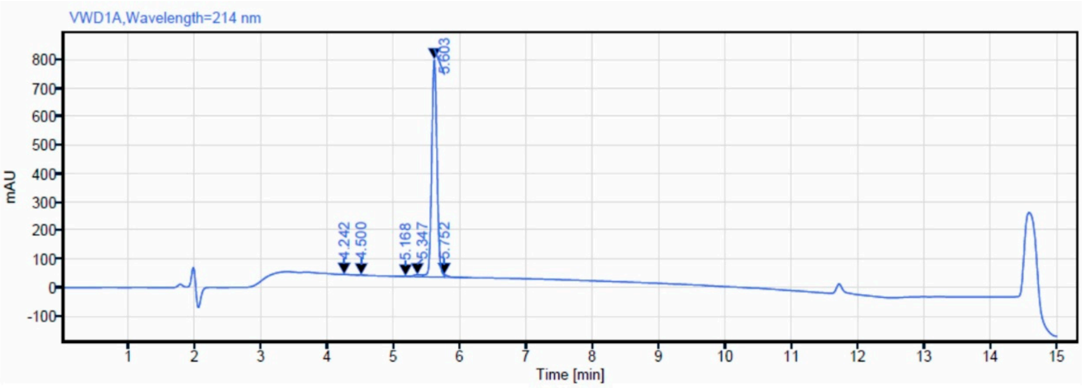

Signal: VWD1A,Wavelength=214 nm

RT [min]	Type	Width [min]	Area	Height	Area%	Name
4.242	MM m	0.24	4.45	0.62	0.10	
4.500	MM m	0.18	6.13	1.43	0.14	
5.168	MM m	0.24	11.68	1.39	0.27	
5.347	MM m	0.18	59.47	7.07	1.38	
5.603	MM m	0.33	4207.14	764.73	97.27	
5.752	MB m	0.35	36.38	8.98	0.84	
		**Sum**	**4325.26**			

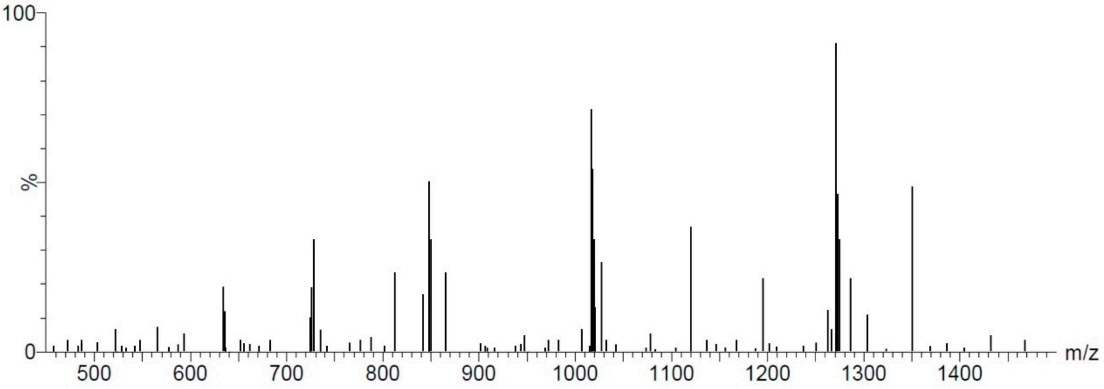

Figure S1. HPLC purity and LC–MS characterization of FMM-66.

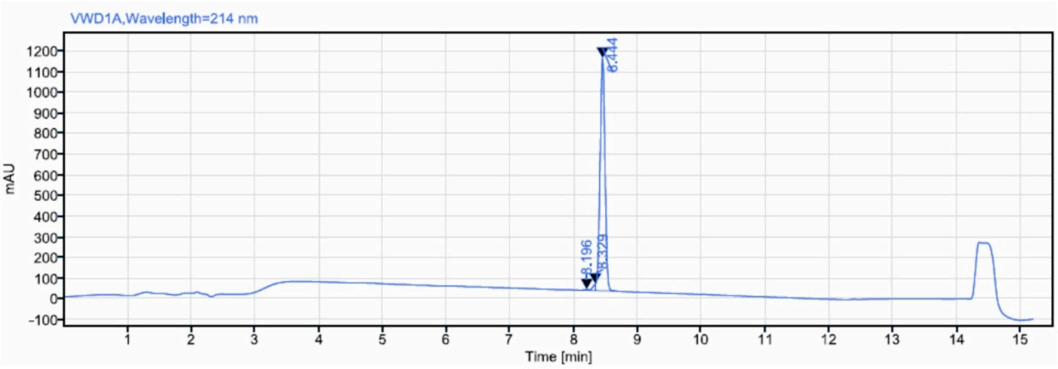

Signal: VWD1A,Wavelength=214 nm

RT [min]	Type	Width [min]	Area	Height	Area%	Name
8.196	MM m	0.10	13.49	3.14	0.22	
8.329	MM m	0.10	87.36	31.24	1.43	
8.444	MM m	0.32	5993.31	1122.42	98.35	
		**Sum**	**6094.16**			

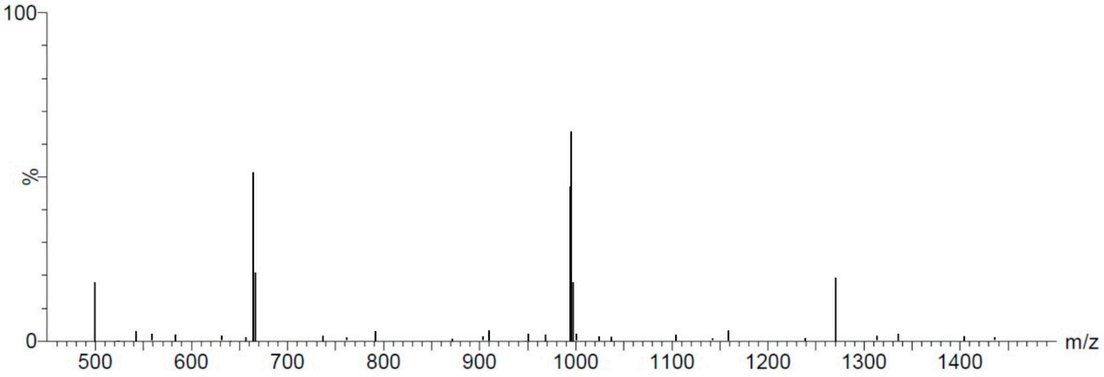

Figure S2. HPLC purity and LC–MS characterization of FA-MMAE.

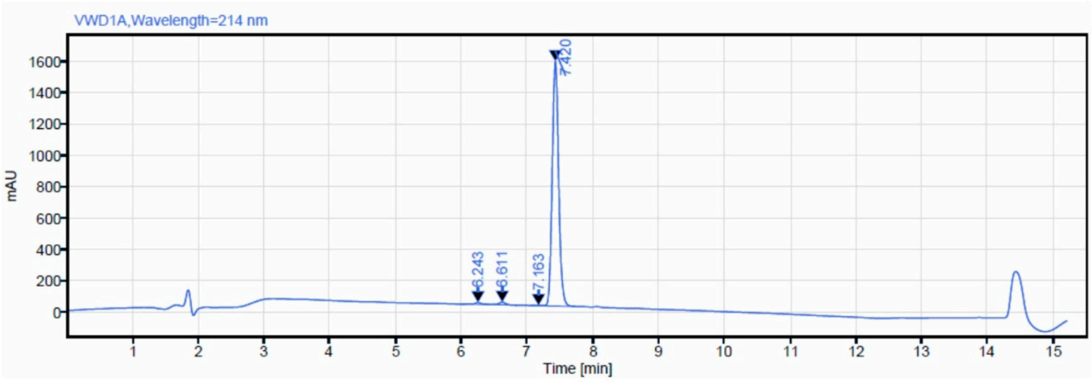

Signal: VWD1A,Wavelength=214 nm

RT [min]	Type	Width [min]	Area	Height	Area%	Name	Peak Relative Ret Time
6.243	MM m	0.44	82.85	11.30	0.77		
6.611	MM m	0.36	108.57	16.61	1.01		
7.163	MM m	0.19	12.79	1.80	0.12		
7.420	VM m	0.49	10555.30	1563.27	98.10		
		**Sum**	**10759.52**				

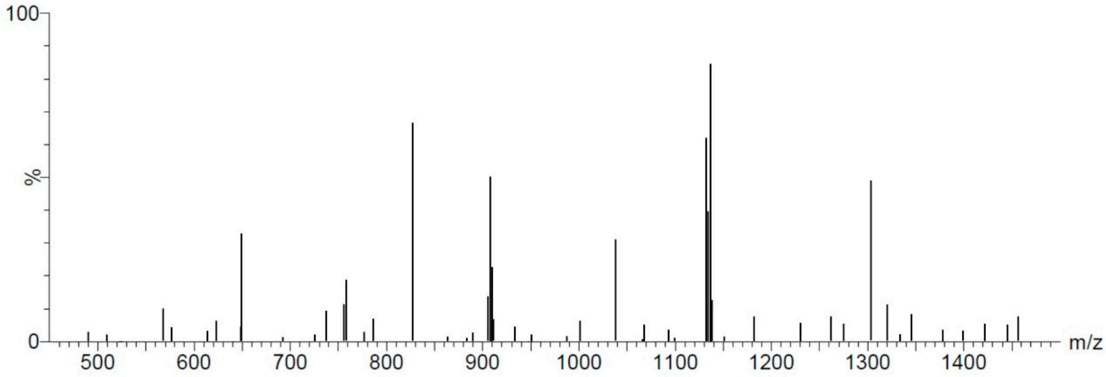

Figure S3. HPLC purity and LC–MS characterization of GE137-MMAE.

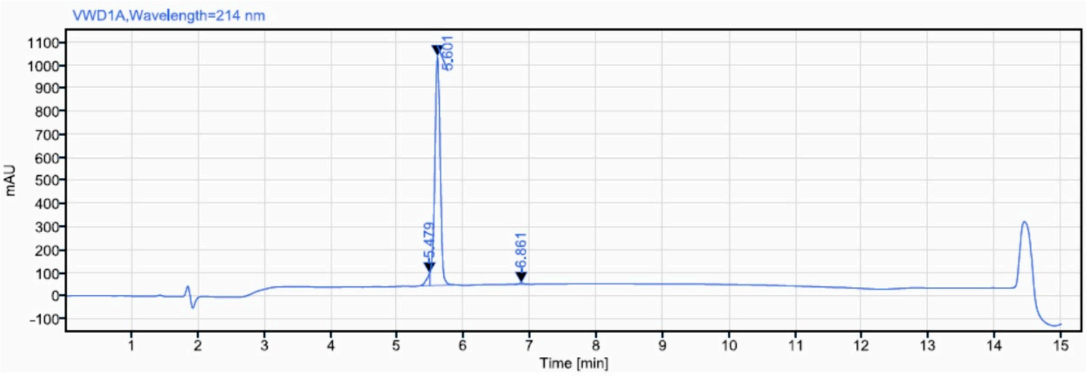

Signal: VWD1A,Wavelength=214 nm

RT [min]	Type	Width [min]	Area	Height	Area%	Name
5.479	MM m	0.16	183.66	52.88	3.13	
5.601	MM m	0.39	5646.87	992.23	96.37	
6.861	MM m	0.20	29.34	5.42	0.50	
		**Sum**	**5859.87**			

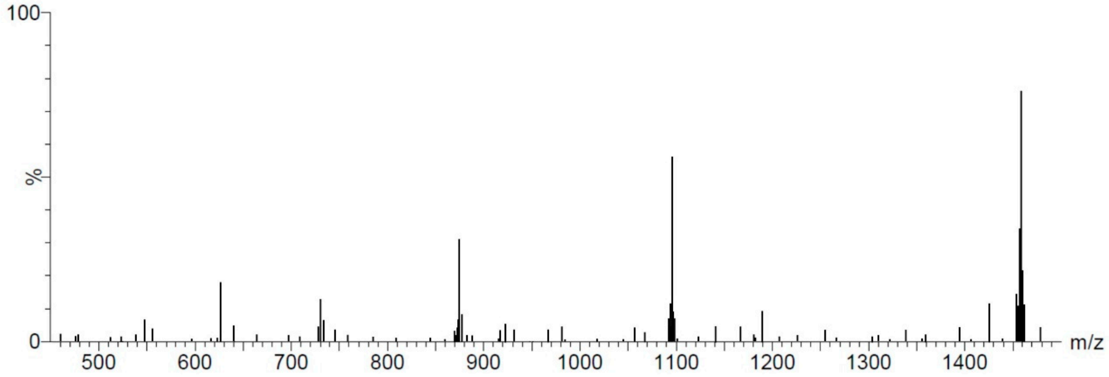

Figure S4. HPLC purity and LC–MS characterization of FMM-66-Cy5.

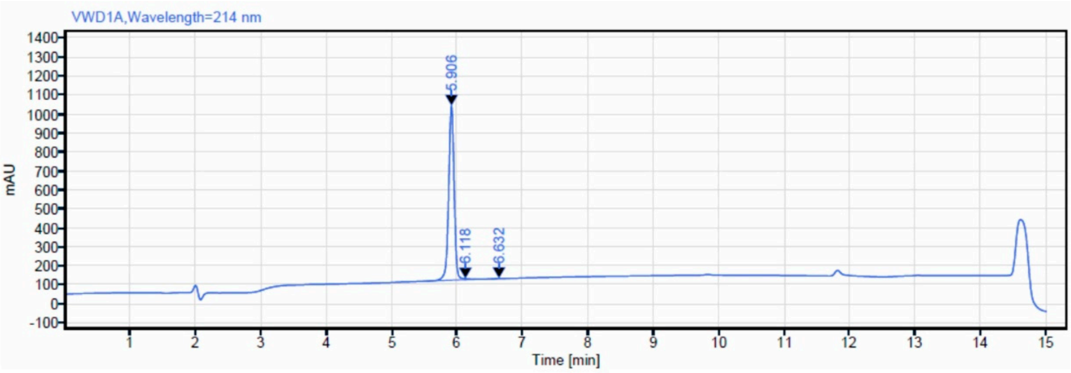

Signal: VWD1A,Wavelength=214 nm

RT [min]	Type	Width [min]	Area	Height	Area%	Name
5.906	BM m	0.59	5332.56	918.90	99.43	
6.118	MB m	0.16	21.70	5.05	0.40	
6.632	MM m	0.28	8.81	1.40	0.16	
		**Sum**	**5363.07**			

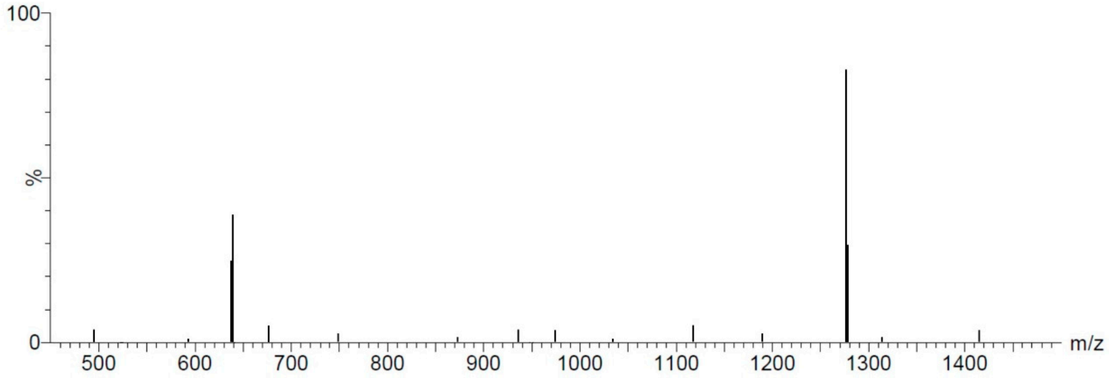

Figure S5. HPLC purity and LC–MS characterization of FA-Cy5.

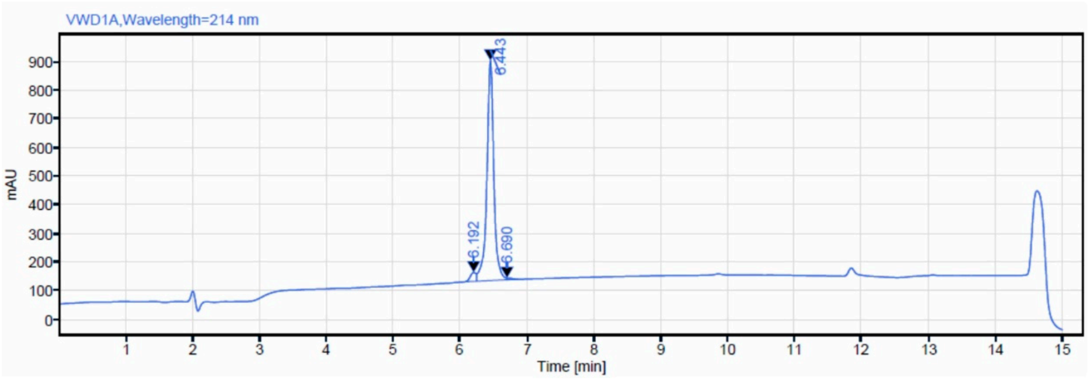

Signal: VWD1A,Wavelength=214 nm

RT [min]	Type	Width [min]	Area	Height	Area%	Name
6.192	MM m	0.17	181.13	30.63	3.15	
6.443	MM m	0.45	5536.77	767.89	96.33	
6.690	MB m	0.17	30.00	5.80	0.52	
		**Sum**	**5747.90**			

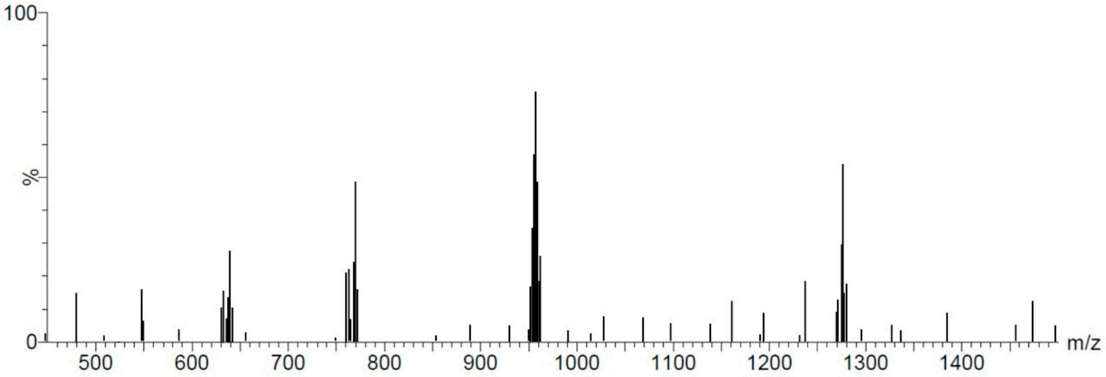

Figure S6. HPLC purity and LC–MS characterization of GE137-Cy5.

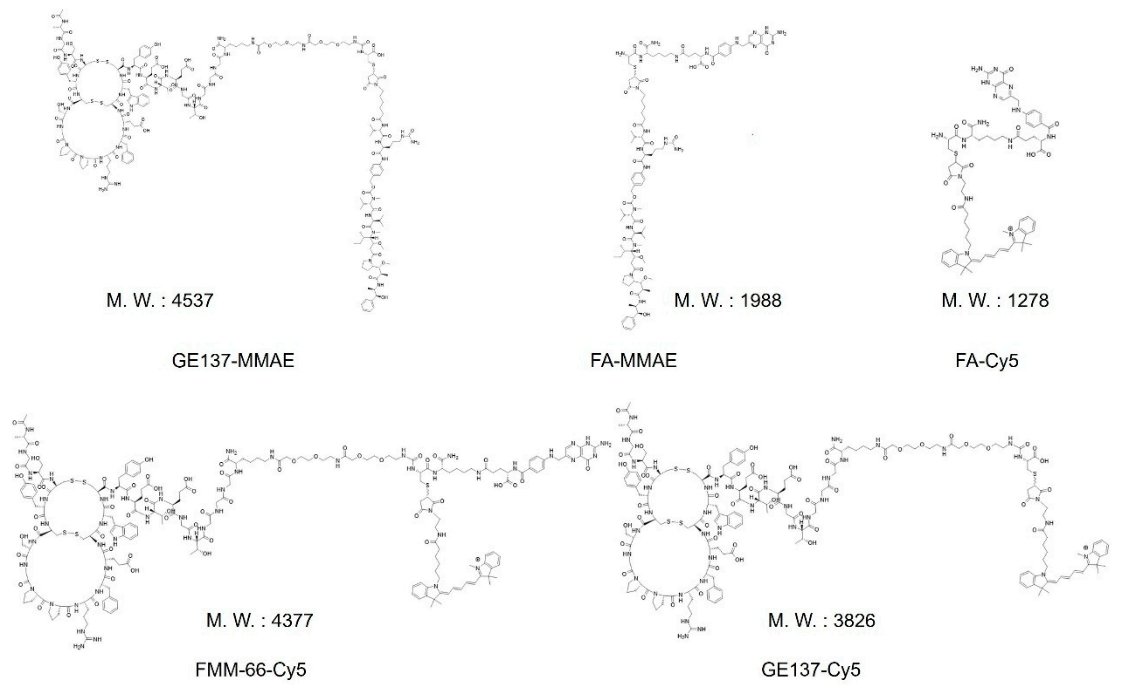

Figure S7. Chemical structure and molecular weight of GE137-MMAE, FA-MMAE, FA-cy5, FMM-66-Cy5 and GE137-Cy5.

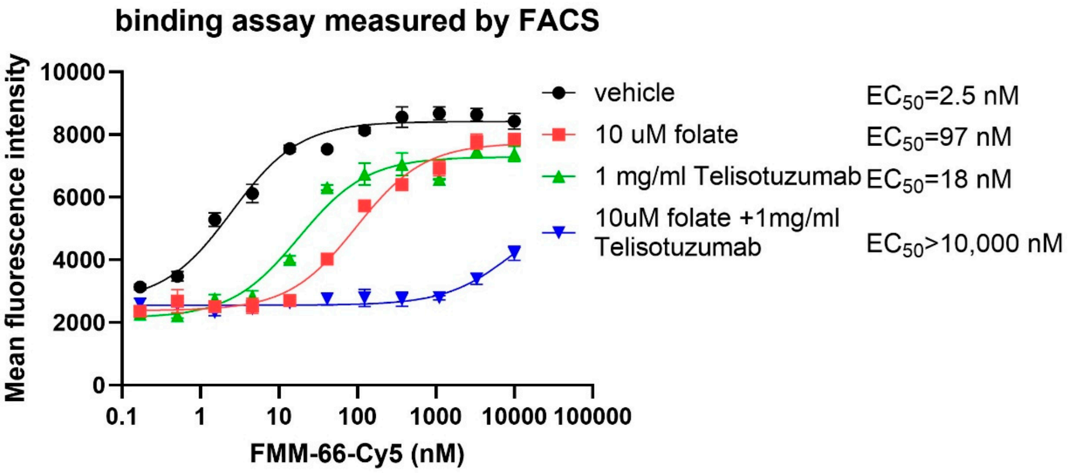

Figure S8. Receptor-blocking analysis of FMM-66-Cy5 binding to FOLR1/c-Met-co-expressing Caov3 cells. Binding of FMM-66-Cy5 to Caov3 cells was evaluated by flow cytometry in the absence or presence of receptor-specific blocking agents. Cells were treated with vehicle, 10 μM folate to block FOLR1, 1 mg/mL telisotuzumab to block c-Met, or a combination of folate and telisotuzumab, followed by incubation with increasing concentrations of FMM-66-Cy5. Cell-associated mean fluorescence intensity was quantified by flow cytometry. Data are presented as mean ± SEM (n = 3 independent biological experiments).

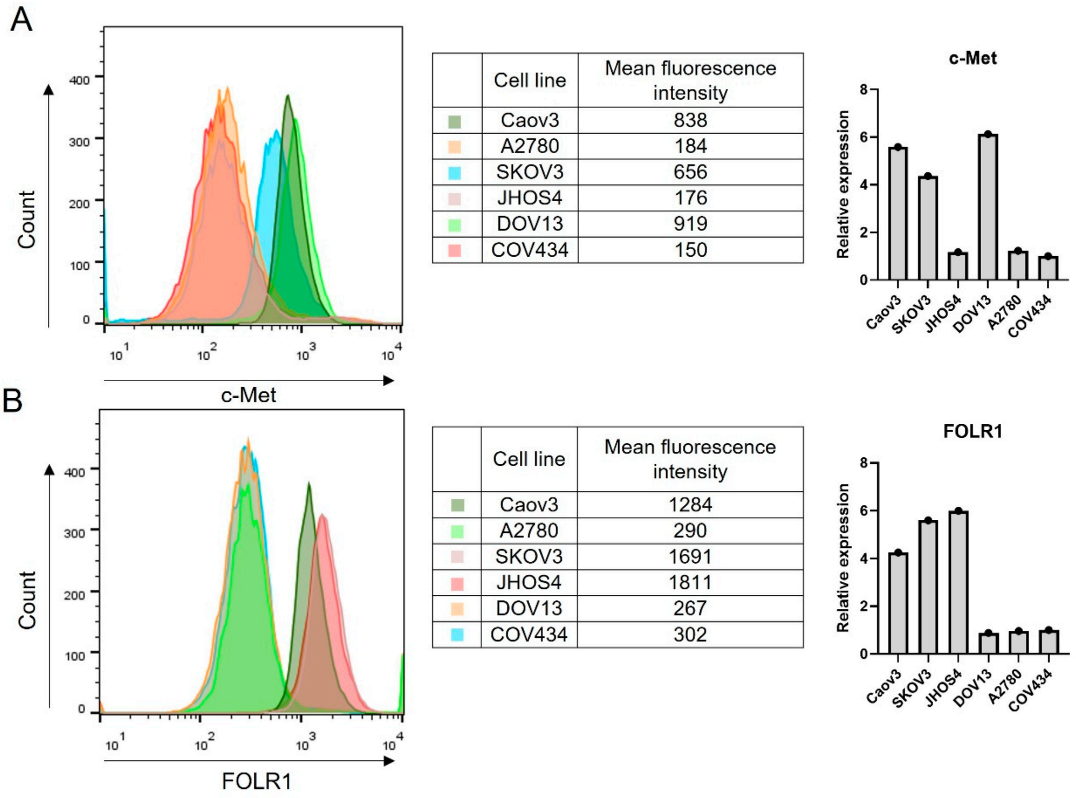

Figure S9. Expression profiles of c-Met and FOLR1 in ovarian cancer cell lines. Surface expression of c-Met and FOLR1 was evaluated by flow cytometry in Caov3, SKOV3, JHOS4, DOV13, A2780, and COV434 cells. (A) Representative overlaid flow cytometry histograms (left) and quantitative analysis of relative c-Met expression (right). (B) Representative overlaid flow cytometry histograms (left) and quantitative analysis of relative FOLR1 expression (right). Mean fluorescence intensity (MFI) values for each cell line are shown in the accompanying tables. Caov3 and SKOV3 cells exhibited relatively high expression of both FOLR1 and c-Met; JHOS4 cells showed high FOLR1 but low c-Met expression; DOV13 cells showed high c-Met but low FOLR1 expression; and A2780 and COV434 cells exhibited relatively low expression of both receptors.

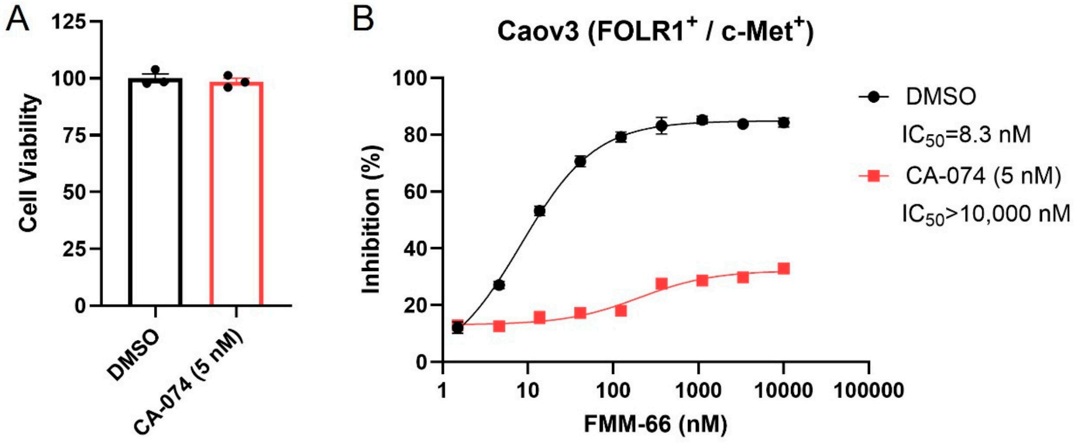

Figure S10. Cathepsin B inhibition attenuates FMM-66-mediated cytotoxicity in Caov3 cells. (A) Effect of the cathepsin B inhibitor CA-074 (5 nM) on the viability of Caov3 cells. (B) Concentration-dependent cytotoxicity of FMM-66 in Caov3 cells in the presence or absence of CA-074 (5 nM). Data are presented as mean ± SEM (n = 3 independent biological experiments).

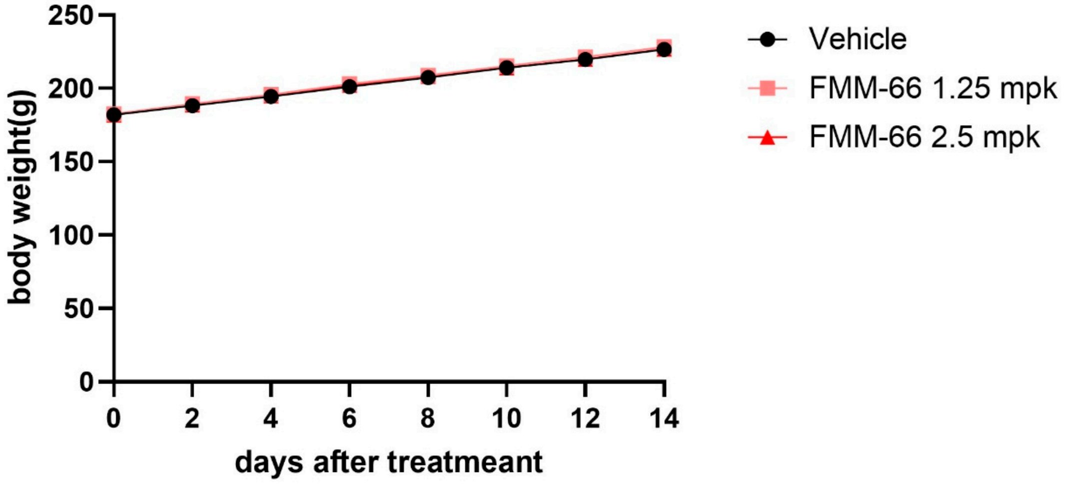

Figure S11. Effect of a single intravenous dose of FMM-66 on body weight in female SD rats. Female SD rats received a single intravenous dose of vehicle or FMM-66 at 1.25 or 2.5 mg/kg. Body weight was monitored for 14 days after administration. Data are presented as mean ± SEM (n = 5 biologically independent animals per group).

Table S1. Pharmacokinetic profiles of intact FMM-66 and the released MMAE in plasma and tumor tissues following a single intravenous administration of FMM-66 (2.5 mg/kg) in Caov3 tumor-bearing mice.

		$T_{1/2}$	T_{max}	C_{max}	AUC_{last}
		(h)	(h)	(ng/mL or g)	(h*ng/mL or g)
FMM-66	plasma	0.75	0.5	7979	2747
	tumor	4.1	1	4228	46100
MMAE	plasma	0.78	0.5	1255	466
	tumor	48.6	6	103.5	4290
